# Dissecting the Molecular Determinants of α‐synuclein Phase Separation and Condensate Aging: The Pivotal Role of β‐Sheet‐Rich Motifs

**DOI:** 10.1002/advs.202511545

**Published:** 2025-09-08

**Authors:** Yiming Tang, Jitao Wen, Zhongyuan Yang, Yifei Yao, Shaoshuai He, Jiyuan Zeng, Xuewei Dong, Qin Qiao, Yun Zhou, Sarah Perrett, Si Wu, Guanghong Wei

**Affiliations:** ^1^ Department of Physics State Key Laboratory of Surface Physics and Key Laboratory for Computational Physical Science (Ministry of Education) Fudan University 2005 Songhu Road, Yangpu District Shanghai 200433 China; ^2^ Key Laboratory of Biomacromolecules CAS Center for Excellence in Biomacromolecules Institute of Biophysics Chinese Academy of Sciences 15 Datun Road, Chaoyang District Beijing 100101 China; ^3^ University of the Chinese Academy of Sciences 19A Yuquan Road, Shijingshan District Beijing 100049 China; ^4^ Center for Soft Condensed Matter Physics and Interdisciplinary Research & School of Physical Science and Technology Soochow University 333 Ganjiang Road, Gusu District Suzhou 215006 China; ^5^ Digital Medical Research Center School of Basic Medical Sciences Fudan University 130 Dongan Road, Xuhui District Shanghai 200032 China; ^6^ Yusuf Hamied Department of Chemistry University of Cambridge Lensfield Road Cambridge CB2 1EW UK

**Keywords:** α‐synuclein, condensate aging, liquid‐liquid phase separation, molecular dynamics simulation, Parkinson's disease

## Abstract

Emerging evidence indicates that liquid‐liquid phase separation of α‐synuclein occurs during the nucleation step of its aggregation, a pivotal step in the onset of Parkinson's disease. Elucidating the molecular determinants governing this process is essential for understanding the pathological mechanisms of diseases and developing therapeutic strategies that target early‐stage aggregation. While previous studies have identified residues critical for α‐synuclein amyloid formation, the key residues and molecular drivers of its phase separation remain largely unexplored. Herein, multiscale simulations and experimental approaches are employed to uncover the molecular determinants dictating α‐synuclein phase separation and the pre‐solidification of its condensates. Seven motifs are identified that exhibit high β‐sheet propensity in the monomeric state of α‐synuclein and progressively increase in β‐sheet content during condensation. Notably, two C‐terminal motifs engage in a percolated network of intermolecular interactions through transient hydrogen bonds, contributing to the phase boundary properties. Deletion of these motifs reduces the phase separation ability of α‐synuclein, underscoring their essential roles in this process. Together, the findings reveal crucial phase separation hotspots and shed light on the molecular mechanism underlying α‐synuclein phase separation, offering significant insights and novel potential therapeutic targets for Parkinson's disease.

## Graphical abstract

Through multiscale simulations and experimental approaches, the molecular determinants of α‐synuclein phase separation and condensate aging, which are linked to Parkinson's disease, are uncovered. Seven motifs are identified with high β‐sheet propensity in monomeric α‐synuclein and increasing β‐sheet tendencies during condensation and pre‐solidification. Persistent and transient interactions among them drive the fibrilization and phase separation of α‐synuclein.

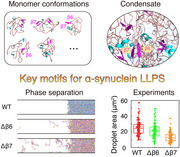

## 1 Introduction

Biomolecular condensates formed through liquid‐liquid phase separation (LLPS) play pivotal roles in the regulation of various cellular functions.^[^ ^1^ ^]^ The dynamic nature of these condensates allows for rapid component exchanges with the cellular environment, enabling precise control of biochemical reactions and regulation of signaling pathways.^[^ ^2^ ^]^ LLPS has also been implicated in the pathogenesis of human diseases, including cancer, inflammatory conditions, and neurodegenerative disorders.^[^ ^3^ ^]^ In recent years, an increasing number of proteins associated with neurodegenerative diseases have been found to undergo LLPS, forming condensates that progressively mature into solid‐like fibrils linked to neuronal death.^[^ ^4^ ^]^ α‐synuclein (αSyn), an 140‐residue intrinsically disordered protein (IDP), is central to the pathology of Parkinson's disease (PD) and other synucleinopathies due to its pathological aggregation into amyloid fibrils, the main component of Lewy bodies.^[^ ^5^ ^]^ Recent studies have demonstrated that αSyn undergoes LLPS both in vitro and in cells,^[^ ^6^ ^]^ and the LLPS of αSyn serves as a precursor to PD‐related pathological aggregation.^[^ ^6^, ^7^ ^]^ Understanding the molecular determinants of αSyn LLPS is crucial for elucidating the mechanisms of LLPS‐mediated aggregation and the pathogenesis of PD and will provide potential targets for therapeutic strategies.^[^ ^6c^ ^]^

The fibrilization of αSyn has been extensively studied, offering valuable insights into the pathological aggregation mechanism.^[^ ^8^ ^]^ A number of fibril structures of full‐length and truncated αSyn have been resolved,^[^ ^9^ ^]^ with various aggregation‐prone regions identified. For example, the preNAC and NACore motifs (residues _47_GVVHGVATVA_56_ and residues _68_GAVVTGVTAVA_78_) play critical roles in the aggregation and cytotoxicity of full‐length αSyn and can individually form amyloid fibrils.^[^ ^10^ ^]^ Residues _36_GVLYVGS_42_ (P1),^[^ ^11^ ^]^_45_KEGVVHGVATVAE_57_ (P2),^[^ ^11^ ^]^ and _2_DVFMKG_7_,^[^ ^12^ ^]^ have been identified as critical segments for αSyn aggregation, whereas removal of these residues prevents αSyn aggregation and suppresses in vivo toxicity. In contrast, the phase separation mechanism of αSyn remains poorly understood, and the specific hotspots driving αSyn LLPS have yet to be identified. Experimental studies have suggested that αSyn phase separation is mediated by an interplay of electrostatic and hydrophobic interactions.^[^ ^6a^ ^]^ Elevating salt concentrations enhances αSyn LLPS, implicating electrostatic screening in this process.^[^ ^6^, ^13^ ^]^ However, the underlying physical forces driving αSyn LLPS remain unclear. In addition, while some aggregation‐promoting factors (e.g., low pH, mutations) have been shown to enhance LLPS and liquid‐to‐solid transitions of αSyn,^[^ ^6a^ ^]^ LLPS of αSyn exhibits a monotonic salt dependence, unlike the non‐monotonic behavior of its aggregation.^[^ ^13b^ ^]^ These discrepancies underscore the urgent need for in‐depth investigation to identify the molecular determinants and hotspots governing αSyn phase separation.

In the mechanistic exploration of protein phase separation, computer simulation has become increasingly prevalent and has achieved significant success in characterizing the phase behaviors of a large number of IDPs and revealing the underlying molecular mechanism.^[^ ^4^, ^14^ ^]^ Theoretic phase simulations have been conducted on various model proteins to reveal their LLPS capabilities.^[^ ^15^ ^]^ Coarse‐grained simulations have been widely employed to examine the phase behavior of IDPs or low sequence complexity domains (LCD) of amyloid proteins.^[^ ^14^, ^16^ ^]^ All‐atom simulations have been conducted on IDP monomers, with their results compared with coarse‐grained simulations and experimental results, to assess the relationship between monomer conformation and LLPS behavior.^[^ ^17^ ^]^ Each of these approaches provides unique insights into the nature of phase separation at specific scales. For IDPs with high sequence complexity, such as αSyn, a comprehensive understanding of the driving forces behind their phase separation and aggregation necessitates the integration of simulation approaches with multiple resolutions alongside experimental data.^[^ ^18^ ^]^

**Figure 1:**
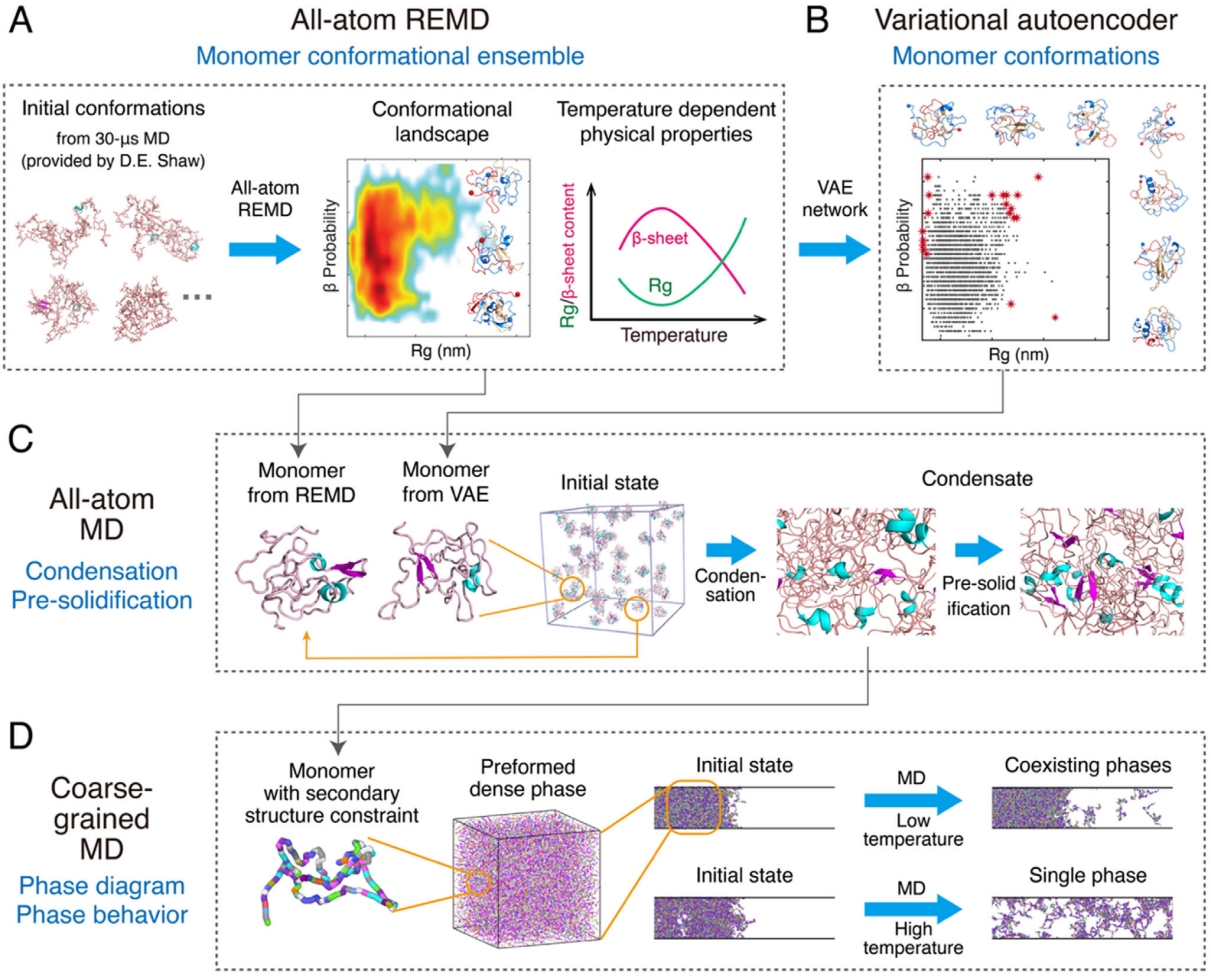
A flowchart illustrating our multiscale simulation approach. A) An all‐atom replica exchange molecular dynamics (REMD) simulation on αSyn monomer with initial structures extracted from a previous 30‐µs MD trajectory.^[^ ^19^ ^]^ B) A variational autoencoder designed to expand the monomer conformational space sampled by the REMD simulation. C) An all‐atom molecular dynamics (MD) simulation on the condensation process of 60 αSyn chains and the subsequent pre‐solidification of the condensate. The initial conformations of the chains were taken from the all‐atom REMD monomer simulation. D) Multiple coarse‐grained (CG) simulations aiming at revealing the phase diagram and phase behavior. The β‐sheet secondary structures of the chains were constrained according to all‐atom simulation data.

In this study, we integrated multiscale simulations with experimental approaches to achieve a comprehensive understanding of αSyn in its monomeric and condensed states. We aimed to identify the molecular determinants and critical residues driving αSyn phase separation by addressing the following unresolved questions: **a)** which physical interactions drive αSyn LLPS; **b)** how the αSyn condensate ages at the atomic level; and **c)** which residues or motifs are critical for these processes? Specifically, we explored αSyn monomer conformations through replica exchange molecular dynamics (REMD) simulation (**Figure** **1**A) with its sampled conformational space expanded by a variational autoencoder (VAE) neural network (Figure 1B), investigated the condensation process through million‐atom molecular dynamics (MD) simulation (Figure 1C), and characterized the phase diagram and phase behavior through coarse‐grained simulations (Figure 1D). These multiscale simulations employed progressively increasing system sizes, with αSyn conformations of each simulation initialized from the preceding one (Figure 1; Table S1, Supporting Information). They elucidate the physical and dynamical properties of αSyn in monomeric and condensed states and provide an atomistic‐resolution understanding of the phase separation pathway. Importantly, we identified seven motifs that exhibit high β‐sheet contents in the monomeric state of αSyn and show an increasing probability of β‐sheet formation during condensation and pre‐solidification. Among them, five motifs provide persistent intermolecular interactions crucial for αSyn aggregation, and two C‐terminal motifs offer transient interactions contributing to the phase boundary properties of αSyn phase separation. The computational predictions were validated by comparing them with existing experiments and our experimental observations on the LLPS and/or fibrillization capabilities of motif‐deleted αSyn variants.

## 2 Non‐Monotonic Temperature Dependency of α‐synuclein Single‐Molecule Properties

**Figure 2:**
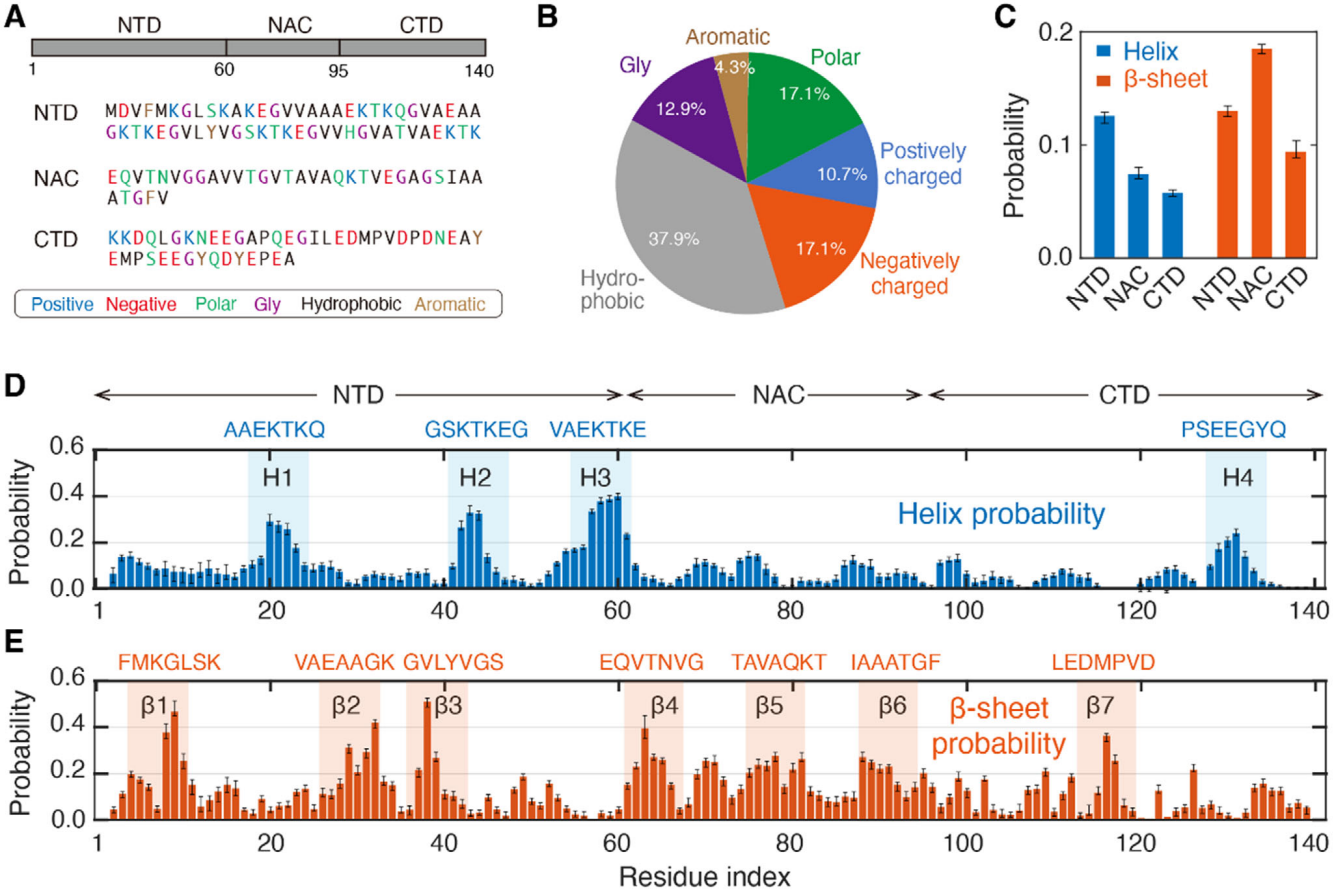
Sequence and secondary structure features of monomeric αSyn. A) Amino acid sequence of αSyn with residues color‐coded based on their physical properties. B) Compositional statistics showing the enrichment of polar and charged residues (44.9% in total). C) Helix and β‐sheet probabilities across the three domains. D,E) Secondary structure propensities at the residue level: (D) helical propensities with helix‐rich motifs highlighted by blue shading and (E) β‐sheet propensities with β‐sheet‐rich motifs highlighted by orange shading.

We began by examining the conformational and physical properties of the αSyn monomer in order to establish a robust foundation and provide a structural basis for detailed investigations into the complex phenomena of phase separation. The 140‐residue αSyn is notably enriched with polar and charged residues (44.9% in total, **Figure** **2**A,B). It comprises N‐ and C‐terminal domains (NTD and CTD) and a non‐amyloid‐β component (NAC), which experimental studies have shown to be crucial for αSyn aggregation.^[^ ^20^ ^]^ We studied the full‐length αSyn monomer by conducting a 400‐ns all‐atom REMD simulation with 60 temperature replicas spanning a range of 308 to 410 K (Table S2, Supporting Information). The protein was described using the Amber99SB‐ILDN force field, which has been used in previous computational studies on αSyn.^[^ ^21^ ^]^ A detailed discussion on the force field selection is provided in Supplementary Text S1, Figures S1–S3, and Table S3 (Supporting Information). We first assessed the convergence and sampling effectiveness of our simulations. The convergence was confirmed by the consistent distributions of radius of gyration (Rg), solvent‐accessible surface area (SASA), and contact numbers within two non‐overlapping time windows (Figure S4, Supporting Information). The sampling effectiveness was evaluated using root‐mean‐square deviation (RMSD), Rg and clustering analyses as described below. Backbone RMSD values between frame pairs show a wide distribution with a maximum value exceeding 2.0 nm (Figure S5A, Supporting Information), and increase over time (Figure S5B, Supporting Information). Rg analyses revealed a wide range of αSyn monomer structures from compact (≈1.4 nm) to extended (≈2.0 nm) states (Figure S2 and Text S1, Supporting Information). Clustering based on a backbone RMSD cutoff of 3.0 nm identified 611 distinct clusters in the 310 K trajectory. The central structures of the 20 largest clusters show substantial variation in structural organization (Figure S6, Supporting Information), secondary structure content, and distribution (Figures S6 and S7, Supporting Information). These results demonstrate that our REMD simulation not only converges but effectively samples a broad and diverse ensemble of monomeric αSyn conformations.

We then investigated the structural characteristics of monomeric αSyn and identified a total β‐structure content of 12.9% from the REMD simulation (Table S4, Supporting Information), which aligns well with experimental data obtained from circular dichroism (CD) and Fourier‐transform infrared (FTIR) spectroscopy studies (11% and 15.6%, respectively, Table S4, Supporting Information).^[^ ^22^ ^]^ Distinct structural propensities were observed across different domains, with NTD displaying a high helix propensity and NAC showing significant β‐sheet content (Figure 2C; Table S4, Supporting Information). This is consistent with experimental findings of the high helical propensity in NTD,^[^ ^23^ ^]^ and the view that NAC is crucial to αSyn fibril formation.^[^ ^24^ ^]^ The residue‐based analysis identified four helix‐rich motifs and seven β‐sheet‐rich motifs, denoted as H1‐H4 and β1‐β7 (Figure 2D,E; Table S5, Supporting Information). The roles of these motifs in αSyn LLPS/fibrilization will be discussed in the following sections. Notably, residue L38 exhibits the highest β‐sheet propensity (Figure 2E), reinforcing its critical role in fibril formation as previously identified in an experimental study.^[^ ^25^ ^]^ We also compared our data with nuclear magnetic resonance (NMR) data.^[^ ^26^ ^]^ and found a positive correlation in β‐sheet distribution along the amino acid sequence (see the detailed comparison in Text S2 and Figures S8–S11, Supporting Information). Collectively, these results underscore the suitability of our selected force field and computational approach in capturing the physical properties of αSyn monomer.

**Figure 3:**
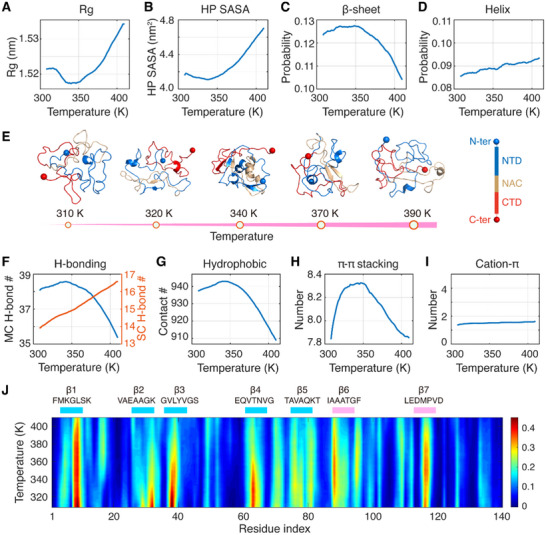
Non‐monotonic temperature dependency of the conformational properties and physical interactions in monomeric αSyn. A–D) Temperature dependency of monomeric physical properties, including (A) Rg, (B) HP SASA, (C) β‐sheet, and (D) helix probability. E) Representative snapshots of αSyn monomer at five temperature points. F–I) Temperature dependency of intrachain physical interactions: (F) MC and SC H‐bonding, (G) hydrophobic, (H) *π–π* stacking, and I) cation‐π interactions. J) β‐sheet propensity across residue indices and temperatures, with seven β‐sheet‐rich motifs highlighted.

Previous studies have shown, for IDPs with low sequence complexity, a close correlation between their single‐chain collapse degree and LLPS capabilities.^[^ ^27^ ^]^ Does a similar correlation exist for αSyn with relatively high sequence complexity? Inspired by previous experiments revealing a non‐monotonic temperature‐dependent phase behavior of αSyn,^[^ ^6a^ ^]^ we monitored the temperature dependence of Rg and the SASA of hydrophobic atoms (HP SASA) of the αSyn monomer. We observed that both parameters exhibit non‐monotonic temperature responses, reaching minimum values at ≈340 K (**Figure** **3**A,B). This behavior was further supported by coarse‐grained simulations using the HPS‐T model, a hydropathy scale force field specifically parameterized to reproduce temperature‐dependent experimental observables of IDPs.^[^ ^28^ ^]^ We also observed a non‐monotonic temperature dependence of Rg in coarse‐grained simulations, but more pronounced compared to all‐atom estimates (Figure S12A,B, Supporting Information), as expected from the model's tuning for capturing temperature‐responsive behavior.^[^ ^28^ ^]^ We then computed the Flory scaling exponent (ν) and observed that αSyn monomers exhibit ν values below 0.5 (signifying sufficient monomer conformational compaction to possess phase separation capability^[^ ^27a^ ^]^) across the 300–380 K temperature range, but display large ν values at both lower and higher temperatures (Figure S12C, Supporting Information). These analyses confirm the non‐monotonic temperature responses of αSyn single‐chain collapse degree.

Additionally, the interplay between secondary structure and the phase behavior of αSyn remains unexplored. Our all‐atom REMD simulation demonstrates that the β‐sheet probability peaks at ≈340 K while the helix probability monotonically increases with rising temperature (Figure 3C,D). These findings indicate that both compactness and β‐sheet (but not helix) content of the αSyn monomer possess non‐monotonic temperature dependences (Figure 3E), resembling the thermoresponsive phase behavior of αSyn.^[^ ^6a^ ^]^ It is thus concluded that the phase separation of αSyn is encoded at the monomeric level, and β‐sheet structures rather than helical structures are important for αSyn LLPS. In our all‐atom REMD simulations, both compactness and β‐sheet probability of αSyn peak at ≈340 K (Figure 3A–C), which exceeds the physiological temperature range. This discrepancy may arise because temperatures in MD simulations do not always directly correspond to experimental temperatures,^[^ ^29^ ^]^ as force fields are usually parameterized to reproduce experimental data at specific temperatures or within limited ranges.^[^ ^30^ ^]^ We thus focus on qualitative trends of αSyn properties in relation to temperature rather than precise temperature values.

## 3 Molecular Determinant Governing the Thermoresponsive Single‐Molecule Properties of α‐synuclein

We elucidated the molecular determinants that govern the thermoresponsive single‐molecule properties of αSyn by analyzing how temperature variations affect the physical interactions within the αSyn monomer. Non‐monotonic temperature dependencies were observed in total contact numbers (Figure S13, Supporting Information), as well as in various physical interactions, including mainchain (MC) hydrogen‐bonding (H‐bonding), hydrophobic and *π–π* interactions (Figure 3F–H). However, no such dependency was observed in sidechain (SC) H‐bonding or cation‐π interactions (Figure 3F–I). These observations reveal that the temperature response of the αSyn monomeric conformational properties is governed by β‐sheet H‐bonding, hydrophobic, and *π–π* interactions. Recent studies have highlighted similarities between intramolecular and intermolecular interactions in IDP systems.^[^ ^31^ ^]^ Thus, although the above analyses are based on a monomer system, they can be extrapolated to phase‐separated systems, namely, intermolecular β‐sheet H‐bonding, hydrophobic, and *π–π* interactions may play crucial roles in the thermoresponsive phase behavior observed in αSyn.^[^ ^6a^ ^]^ This will be further verified through our simulation of αSyn condensation in the subsequent section.

To identify motifs that are critical for the thermoresponsive secondary structure properties of αSyn, we examined the temperature dependency of β‐sheet contents at the residue level. We focus on the seven motifs with high β‐sheet probability under physiological temperature (Figure 2E; Table S5, Supporting Information). Among them, only β6 and β7 display non‐monotonic temperature dependencies in β‐sheet propensity (Figure 3J; Figure S14, Supporting Information) which resemble the thermoresponsive phase behavior of αSyn.^[^ ^6a^ ^]^ Thus, it is likely that β6 and β7 motifs are pivotal for the LLPS of αSyn. Further analysis on sequence characteristics reveals that the β6 motif is enriched with hydrophobic and aromatic residues (_88_IAAA_91_ and F94) that can act as “stickers”, and that the glycine residue G93 may serve as a “spacer” (Figure S15, Supporting Information). This corresponds to the theory that the LLPS of polymers/IDPs is dictated by hydrophobic stickers separated by spacers, which respectively drive inter‐chain interactions and impart monomer flexibility.^[^ ^32^ ^]^ Additionally, the β7 motif is enriched with negatively charged residues (E114, D115, D119) (Figure S16, Supporting Information), which may enhance intermolecular electrostatic interactions between the negatively charged CTD and the positively charged NTD, as also evidenced by the high β7‐β1 and β6‐β1 contact numbers (Figure S17, Supporting Information), and ultimately the LLPS of αSyn. Previous studies have shown that CTD‐mediated electrostatic interactions are major driving forces for the heterogeneous phase separation of αSyn with various protein partners, including Tau,^[^ ^33^ ^]^ PrP,^[^ ^34^ ^]^ and vesicle‐associated membrane protein 2 (VAMP2).^[^ ^35^ ^]^ Given the location of β7 motif within CTD and its enrichment in negative charges, β7 is likely to play a critical role not only in promoting αSyn phase separation but also in facilitating its co‐phase separation with binding partners.

Conversely, the other five motifs (β1‐β5) demonstrate a monotonic decrease in β‐sheet propensity as temperature increases and possess high β‐sheet probability in the physiological temperature range (Figure 3J; Figure S14, Supporting Information). Given that cross‐β‐sheet is a common structural feature of amyloid fibrils,^[^ ^36^ ^]^ these motifs are likely crucial for αSyn fibril formation. In support of our predictions, recent studies have underscored the importance of β3 and a motif overlapping with β5 in the fibrillization of αSyn (Figure S18A–C, Supporting Information).^[^ ^11^, ^37^ ^]^ In addition, β4 and β5 motifs are located within the NAC region (Figure S18A–D, Supporting Information), which is known to play a key role in αSyn aggregation.^[^ ^20^ ^]^ In our simulation, the β1 motif exhibits the highest β‐sheet propensity among all motifs and retains stability over a broad temperature (Figure 3J; Figure S14A, Supporting Information), suggesting its crucial role in αSyn fibrillization. This role was further supported by our ThT fluorescence assays comparing wild‐type αSyn (αSyn_WT_) with variants lacking either the β1 or β3 segment (αSyn_Δβ1_ and αSyn_Δβ3_, respectively). The β1‐deleted variant displays a markedly prolonged lag phase relative to αSyn_WT_ (Figure S19, Supporting Information), and the deletion of the β3 segment also extends the lag phase, though to a lesser extent than β1 deletion (Figure S19, Supporting Information). After completion of our ThT experiments, we noticed a recent study that identifies residues 2–7, which have four residues overlapping with β1 (residues 4–10, Figure S18E, Supporting Information), as regulatory elements for fibrillization in that deletion of these residues slows fibril formation and diminishes the protein's capacity to be recruited by wild‐type fibrils.^[^ ^12^ ^]^ In addition, a very recent study has revealed that truncation of residues 1–18, which include β1, does not affect the ability of αSyn to undergo LLPS but accelerates αSyn amyloid formation.^[^ ^38^ ^]^ These findings collectively confirm the critical role of β1 in αSyn fibrillization.

## 4 Persistent and Transient Interactions that Drive the Spontaneous Condensation of α‐synuclein and the Subsequent Pre‐Solidification

We then investigated the condensation and possible pre‐solidification processes of αSyn using an all‐atom simulation starting from non‐contacting αSyn chains. Most computational studies on LLPS of IDPs, constrained by computational resources, rely on coarse‐graining, which simplifies the system and models only a subset of interactions, posing challenges to the accurate recapitulation of experimentally accessible behaviors.^[^ ^39^ ^]^ Here, we utilized a 3D‐computing facility to conduct a large‐scale, microsecond simulation involving more than one million atoms, starting from 60 non‐contacting αSyn molecules in an aqueous solution. This approach allows for a direct, atomistic‐resolution observation of the spontaneous condensation of αSyn and the time evolution of the condensate. The initial structures of the 60 αSyn molecules (Figure S20, Supporting Information) were distinct from one another, derived from our REMD monomer simulations and further enriched using a VAE‐based generative network trained on the REMD trajectory to expand the sampled conformational space. During the 3‐µs simulation, the time evolutions of backbone RMSD for individual chains exhibit a broad distribution, ranging from ≈0.3 nm to as high as 1.5 nm (Figure S21A, Supporting Information), and Rg of each chain fluctuates markedly over time, with the most pronounced variation spanning from 1.5 to 2.2 nm (Figure S21B, Supporting Information). In addition, both parameters show an overall increasing trend. These results demonstrate that the simulation captures appreciable conformational change of the 60 chains during condensation.

**Figure 4:**
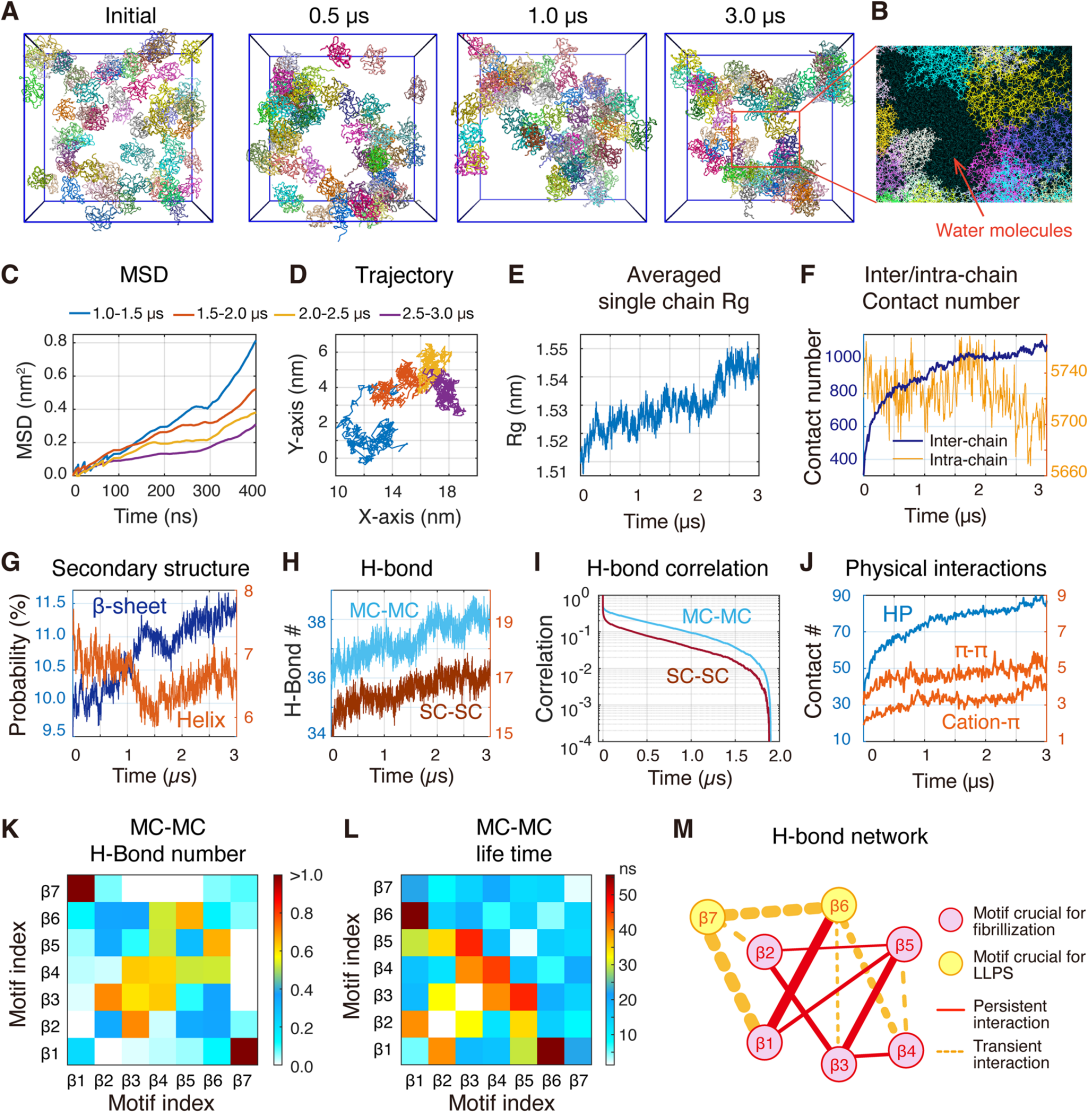
Condensation of 60 αSyn chains and pre‐solidification of the condensate. A) Simulation snapshots at four time points. B) Detailed snapshot within a localized area of the condensate showing the interaction of αSyn chains with water molecules. C) MSD of αSyn molecules as a function of lag time across four distinct time windows. D) Trajectory of the center‐of‐mass for a representative αSyn chain within the condensate, with each of the four time windows highlighted in different colors. E) Time evolution of Rg averaged on 60 αSyn chains. F) Time evolution of intra‐ and inter‐chain contact numbers. G) Evolution of β‐sheet and helix contents over simulation time. H) Time evolution of MC—MC and SC—SC H‐bond numbers. I) Time autocorrelation functions for MC and SC H‐bonds. J) Time evolution of hydrophobic, *π–π*, and cation‐π interactions. K) Number of mainchain H‐bonds between each pair of β‐sheet‐rich motifs. L) Lifetime of MC─MC interactions between each two motifs. M) Illustration of the mainchain interaction network formed by the seven β‐sheet‐rich motifs. Wider lines correspond to motif pairs with higher H‐bond numbers.

We first assessed the condensation pathways via visual inspections. Starting from a randomly dispersed state, the 60 αSyn molecules rapidly aggregated into a loosely packed assembly within 1.0 µs (**Figure** **4**A). Afterward, αSyn molecules continued repositioning within the aggregate (Figure 4A). The aggregate comprises cross‐linked chains interspersed with substantial quantities of water molecules, possessing characteristics of liquid‐like condensate.^[^ ^2a^ ^]^ (Figure 4B; Movie S1, Supporting Information). Mean square deviation (MSD) analysis on the initially formed condensate (Figure 4C) reveals a diffusion coefficient of ≈2.01 × 10^−12^ m^2^ s^−1^ (Table S6, Supporting Information), within the same order of magnitude as both experimentally determined (2.7 × 10^−12^ m^2^ s^−1^) and computationally predicted diffusion coefficients for the dense phase of ProTα (1.8 × 10^−12^ m^2^ s^−1^),^[^ ^40^ ^]^ an IDP with a sequence length (111 aa) similar to αSyn (140 aa). This further demonstrates the liquid properties of αSyn condensate. Afterward, the MSD values decrease over simulation time (Figure 4C), coinciding with the reduction in diffusion constant (Table S6, Supporting Information). These observations suggest a progressive decrease in the molecular mobility of αSyn molecules within the condensates over time (Figure 4D). Such a reduction in mobility implies a gradual loss of condensate liquidity, indicative of pre‐solidification, a potential early step in the aging process. Although the timescale of MD simulations (on the order of microseconds) is insufficient to directly capture the full progression of condensate maturation, the observed molecular rearrangements resemble the early stage of condensate aging reported in various LLPS‐induced IDP condensates.^[^ ^41^ ^]^ Recent studies have uncovered that biological condensates form percolated networks to ensure strong interactions and transient confinement.^[^ ^42^ ^]^ To determine whether the αSyn condensates exhibit characteristics of a percolated network, we analyzed the time evolution of network connectivity,^[^ ^42^ ^]^ a parameter that characterizes the strength of system‐spanning interaction networks, within a cubic 3D model. We observed a gradual increase in network connectivity, reaching an equilibrium value exceeding the percolation threshold of 0.5 (Figure S22, Supporting Information). This suggests that αSyn molecules form a molecular network mediated by multivalent interactions, exhibiting characteristics of percolation.

To elucidate the molecular determinant governing the condensation and pre‐solidification processes, we monitored the time evolution of single‐chain conformations and intra‐/inter‐chain interactions of αSyn molecules. Over time, the Rg of αSyn chains gradually increases (Figure 4E), accompanied by increasing inter‐chain and decreasing intra‐chain contacts (Figure 4F), indicating a progressive elongation and a shift from intra‐chain to inter‐chain interactions. These observations are consistent with recent studies demonstrating elongated conformations upon LLPS in αSyn,^[^ ^43^ ^]^ and other IDPs.^[^ ^44^ ^]^ Concurrently, β‐sheet content rises, while helix content changes slightly (Figure 4G; Figure S23, and Movie S2, Supporting Information), marking the structural rearrangement of the condensate into β‐rich aggregates.

We further analyzed the contribution of different physical interactions in the condensation and pre‐solidification processes. We observed an increase in H‐bond numbers over time, with MC─MC H‐bonds significantly outnumbering and exhibiting higher time correlations than SC─SC H‐bonds (Figure 4H,I). Hydrophobic and *π–π* contacts exhibit a rapid rise while cation‐π contacts remain at the lowest levels (Figure 4J). These findings confirm the dominance of MC β‐sheet H‐bonding, hydrophobic, and *π–π* interactions in driving αSyn LLPS as predicted by our monomer REMD simulation. We next assessed the interactions mediated by the seven β‐sheet‐rich motifs, all of which exhibit relatively high and increasing β‐sheet propensities during condensation (Figure S24, Supporting Information). Strong and persistent MC H‐bonds were observed for pairs involving β3, β4, and β5 motifs, particularly for β3—β4, β4—β4, and β3—β5 pairs (Figure 4K,L). Interactions involving β1 and/or β2 motifs (β1—β2/β3/β5 and β2—β5 pairs) are relatively infrequent (Figure 4K) yet possess long lifetimes once formed (Figure 4L). In contrast, MC interactions involving β6 and/or β7 motifs are mostly transient (Figure 4L), indicating a dynamic association. Notably, strong intermolecular H‐bonds form between β1 and β7. This observation, together with the pronounced intramolecular interaction between β1 and β7 observed in our monomer simulations (Figure S17, Supporting Information), suggests that electrostatic interactions between the N‐ and C‐terminal domains of αSyn are preserved during condensation but are repurposed from intra to intermolecular contacts. Interestingly, despite being the strongest interaction among all motif pairs, the β1—β7 interaction remains relatively transient, further highlighting the critical role of β7 in phase separation.

Existing literature emphasizes that a delicate balance between persistent and transient intermolecular interactions is crucial for biomolecular phase separation.^[^ ^17^, ^40^ ^]^ These interactions must be strong enough to facilitate the formation of stable condensates yet sufficiently weak to allow for translational diffusion and liquid‐like dynamics within the dense phase.^[^ ^2^, ^3^, ^40^ ^]^ In the case of αSyn, the seven β‐sheet‐rich motifs form a tight, interconnected H‐bond network (Figure 4M). Specifically, the β1‐β5 motifs provide persistent H‐bonds that constitute the skeleton of the interaction network, and β6/β7 motifs engage in the network through frequent but transient interactions.

## 5 Computational and Experimental Verification of the Impairment of α‐synuclein LLPS by β6/β7 Deletion

**Figure 5:**
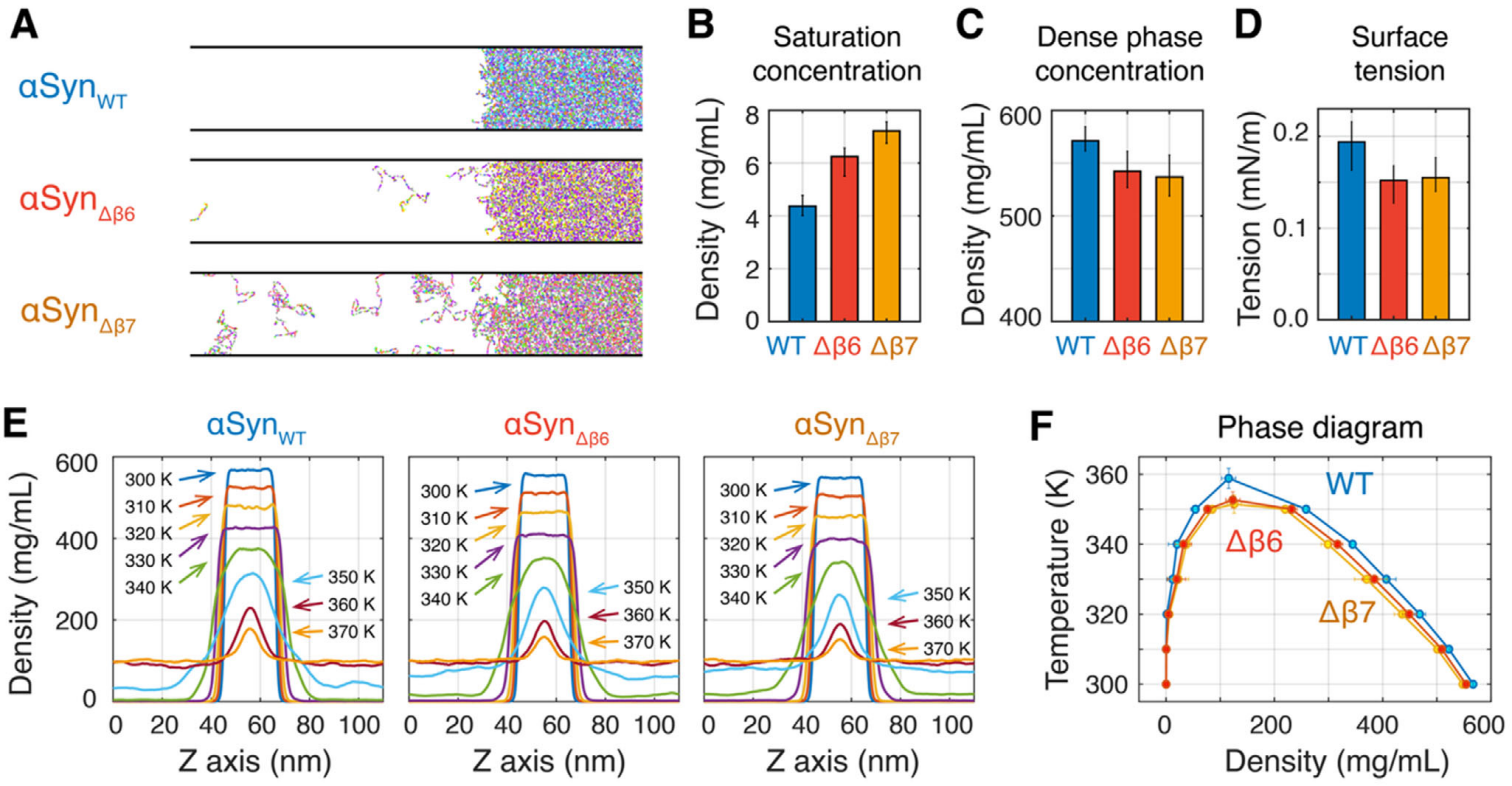
Coarse‐grained phase coexistence simulations unraveling the critical role of β6 and β7 motifs on αSyn LLPS. A) Final snapshots of the phase coexistence simulations. For clarity, only part of the simulation box is shown. B) Saturation concentration of the three systems. C) Dense phase density of the three systems. D) Surface tension of the interface between dense and dilute phases. E) Density profiles of the three systems. F) The phase diagrams as a function of density and temperature.

To validate the critical role of β6 and β7 motifs in αSyn LLPS, we conducted coarse‐grained simulations and in vitro experiments on αSyn and its motif‐deleted variants. For each system, phase coexistence simulations were performed in combination with the hydropathy scale (HPS) protein model^[^ ^16b,c^ ^]^ to generate an equilibrated coexistence state comprising a dense phase and a dilute phase (**Figure** **5**A). The dense phase was then placed in a cubic box and simulated to assess its physical properties.^[^ ^14c^ ^]^ To account for secondary structures that are not included in the HPS force field,^[^ ^16e^ ^]^ we integrated the conformational characteristics of αSyn revealed by all‐atom simulations into the original HPS framework by introducing additional harmonic angle terms for each set of three adjacent residues within the β‐sheet‐rich motifs. The parameters for these terms were assigned based on the β‐propensities of each residue, as determined by our all‐atom condensation simulations (see details in the Methods section). This modification enables an extended yet flexible conformation of the β‐sheet‐rich motifs (Figure S25, Supporting Information), with their degree of extension correlating with their β propensities (Figure S26, Supporting Information).

Our simulations at physiological temperature revealed that deleting the β6 or β7 motif (αSyn_Δβ6_ and αSyn_Δβ7_) increases the saturation concentration while reducing the density of the dense phase (Figure 5B,C), indicating the reduced LLPS capabilities due to motif deletion. The surface tension at the two‐phase interface decreases upon motif deletion (Figure 5D), suggesting the weakening of condensate stability.^[^ ^45^ ^]^ We further explored the temperature‐dependent phase separation by conducting phase coexistence simulations at a series of temperatures. For each system, the dense phase disperses progressively with increasing temperature, culminating in a complete loss of phase separation capability at high temperatures (Figure 5E; Movie S3, Supporting Information). At each temperature point within the phase separation regime, both αSyn_Δβ6_ and αSyn_Δβ7_ consistently exhibit lower dense‐phase densities and higher dilute‐phase densities compared to αSyn_WT_ (Figure 5E; Movie S4, Supporting Information). Phase diagrams (Figure 5F) reveal lower critical temperatures for αSyn_Δβ6_ (352.6 ±2.3 K) and αSyn_Δβ7_ (351.4 ±2.6 K) versus αSyn_WT_ (358.8 ±2.8 K). These results demonstrate that the deletion of β6 and β7 decreases the phase separation capability of αSyn and destabilizes its condensate, confirming the importance of β6 and β7 in αSyn LLPS.

The importance of β‐structures on phase behavior was further assessed by comparing these results with simulations using the original HPS force field, which lacks secondary structure constraints for the β‐sheet‐rich motifs. The removal of these constraints led to a marked decrease in the critical temperature and the density of the dense phase (Figure S27, Supporting Information), indicating a reduced phase separation capability. Moreover, the effect of β6 or β7 motif deletion on phase separation remains robust regardless of the inclusion or exclusion of the angle term. These findings further underscore the critical role of the identified β‐sheet‐rich motifs in promoting αSyn phase separation.

While previous experimental studies have demonstrated that αSyn exhibits both lower and upper critical solution temperature (LCST and UCST) behaviors,^[^ ^6^, ^46^ ^]^ our simulations captured only the UCST behavior. This limitation arises from the lack of temperature‐dependent solvent interactions in the HPS protein force field.^[^ ^28^ ^]^ We thus performed additional simulations of αSyn_WT_ at three temperatures using the HPS‐T force field, which incorporates temperature‐dependent interactions.^[^ ^28^ ^]^ These simulations reveal that αSyn undergoes LLPS at physiological temperature (310 K), but not at either lower (280 K) or higher (390 K) temperatures (Figure S28, Supporting Information), indicative of both UCST and LCST phase behaviors.

**Figure 6:**
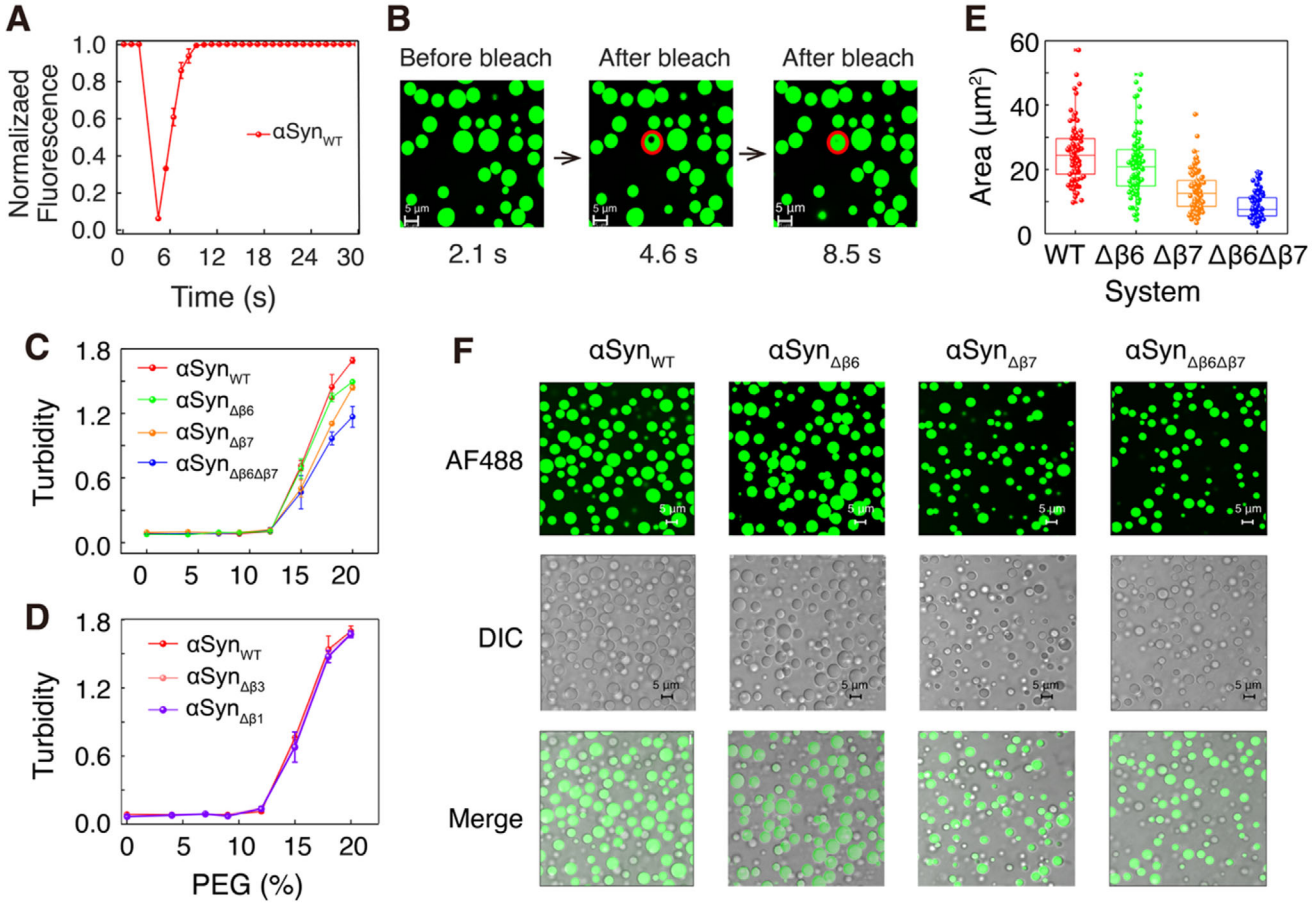
Experimental characterization of the phase behavior of αSyn and its β6, β7, and β6+β7 deletion variants. A) Time‐dependent fluorescence intensity trace of Alexa488‐labeled αSyn in the FRAP experiment. B) FRAP images of Alexa488‐labeled αSyn droplets before and after photobleaching. A region in the droplet, marked by the red circle, was bleached. C) Turbidity assays of αSyn_WT_ and its variants (αSyn_Δβ6_, αSyn_Δβ7,_ and αSyn_Δβ6Δβ7_) in the presence of different concentrations of PEG measured at 400 nm. D) Turbidity assays of αSyn_WT_ and its variants αSyn_Δβ3_ and αSyn_Δβ1_. E) Area statistics of the droplets formed by αSyn and its variants. For each variant, 90 droplets were counted. F) Fluorescence and DIC images of the droplets formed by αSyn_WT_ and its variants (unlabeled αSyn doped with 10% Alexa488‐labeled αSyn).

To further verify our computational findings, we experimentally constructed αSyn mutants with the deletion of either β6 or β7 or β6+β7 (αSyn_Δβ6_, αSyn_Δβ7_, and αSyn_Δβ6Δβ7_) and purified the proteins. In the presence of PEG as a crowding reagent mimicking the crowded cellular environment, spherical droplets of micrometer size were observed for αSyn protein. The liquid‐like properties of droplets were tested by fluorescence recovery after photobleaching (FRAP) experiments (**Figure** **6**A,B). The droplets formed by unlabeled αSyn, doped with 10% Alexa488‐labeled αSyn, show rapid fluorescence recovery after photobleaching, indicating liquid‐like properties. The droplets formed by the variant αSyn_Δβ6Δβ7_ also showed similar behavior (Figure S29, Supporting Information). These results demonstrate that both αSyn_WT_ and the deletion variants can form liquid droplets under our experimental conditions. By fitting the fluorescence recovery curves to a single‐exponential function, we obtained a recovery half‐time of 0.75±0.02 s for the β6/β7 deletion mutant, which is comparable to that of wild‐type αSyn (0.74±0.02 s). This result suggests that deletion of the β6/β7 motifs does not substantially alter the internal dynamics or molecular exchange rates within the condensates. Instead, they primarily affect the phase boundary properties of αSyn. We then carried out turbidity assays to compare the LLPS capability of αSyn_WT_ and its deletion variants. The three variants show reduced turbidity values at a PEG concentration above 12%, and this reduction in turbidity is more pronounced for αSyn_Δβ6Δβ7_ (Figure 6C). As a control, we also carried out turbidity assays for αSyn_Δβ1_ and αSyn_Δβ3_ (with deletion of the β1 or β3 motif), which our simulations predict to be unrelated to αSyn LLPS. Both αSyn_Δβ1_ and αSyn_Δβ3_ show a comparable turbidity value to αSyn_WT_ (Figure 6D), indicating that these two motifs have little effect on the LLPS of αSyn.

Additionally, we performed fluorescence and DIC imaging of the droplets formed by Alexa488‐labeled αSyn_WT_ and its deletion variants (Figure 6E,F). The droplets formed by αSyn_Δβ6Δβ7_ appear to be smaller and less abundant compared to those formed by αSyn_WT_. The statistics of the droplet area revealed that all three deletions (β6, β7, and β6+β7) lead to a reduction in droplet size, albeit with different extents of size reduction. Those results, together with the turbidity assays, demonstrate that the deletion of β6, β7, or β6+β7 impairs the LLPS capability of αSyn. We also checked the temperature effect on the LLPS of αSyn_WT_ and the β6+β7 deletion variant αSyn_Δβ6Δβ7_ by imaging (Figure S30, Supporting Information). The results show that the formation of αSyn_WT_ droplets exhibits a non‐monotonic temperature‐dependent behavior consistent with previous reports.^[^ ^6a^ ^]^ In contrast, the LLPS of αSyn_Δβ6Δβ7_ nearly loses sensitivity to temperature, a phenomenon that remains to be further investigated. Altogether, these experimental results demonstrate that β6 and β7 motifs are critical for the phase behavior of αSyn, in support of our simulation predictions.

## 6 Discussion

One major challenge in studying IDPs is their inherent lack of persistent structures,^[^ ^39^, ^47^ ^]^ i.e., their extensive array of metastable states.^[^ ^39^, ^48^ ^]^ This complexity poses significant difficulties for conventional MD simulations, which often suffer from insufficient sampling,^[^ ^49^ ^]^ and tend to trap the system in metastable states for extended periods.^[^ ^49a^ ^]^ Specifically, αSyn monomer has been extensively simulated by D.E. Shaw and coworkers^[^ ^19^ ^]^ using various force fields, including Amber99SB*‐ILDN and two IDP‐specific force fields, CHARMM36m and Amber99SB‐disp. These simulations exhibited a progressive decrease in inter‐frame backbone RMSD values over time and a continuous decline in the average RMSD of each frame relative to all others (Figure S31A–F, Supporting Information), indicating limited and decreasing structural variability. These observations suggest that, despite the long duration of conventional MD simulations, the system remains confined to local energy minima and fails to adequately explore the diverse conformational ensemble of αSyn. In addition, AI‐based structure prediction tools designed for structured proteins cannot capture the multi‐conformational nature of IDPs,^[^ ^50^ ^]^ as seen with AlphaFold 2, which consistently predicts a monomeric αSyn configuration with a long helical N‐terminal (Figure S32, Supporting Information), corresponding to a membrane‐bound state.^[^ ^51^ ^]^ This study addresses this challenge using a long all‐atom REMD simulation in combination with a VAE conformation generator, providing a comprehensive and accurate depiction of the conformational space. In sharp contrast to conventional MD, the inter‐frame RMSD values from our REMD simulations increase over time (Figure S31G,H, Supporting Information), demonstrating that the system escapes from local minima and continues to explore a wide range of conformations throughout the simulation.

However, such a long REMD simulation is notably time‐consuming, requiring ≈200 days on a GPU card. Recognizing the need for more efficient methods, we explored the use of a generative autoencoder.^[^ ^52^ ^]^ The strength of the VAE network was assessed by training it on the first half of our REMD trajectory, and the trained network is capable of producing conformations not present in the training set but observed in subsequent parts of the trajectory (Figures S33 and S34, Supporting Information). The conformational landscape generated by the VAE closely resembles that sampled by REMD, particularly in terms of the locations of major energy minima (Figure S34A,B, Supporting Information). However, at the periphery of the sampled conformational space, the VAE enables an expansion beyond the boundaries explored by REMD, capturing novel conformations that are absent from the REMD ensemble. This highlights the VAE's potential to enhance structural diversity by efficiently sampling low‐probability but physically plausible states. Interpolating within the latent space produced 10000 samples whose β‐sheet distribution closely matches our 400‐ns REMD simulation (Figure S35, Supporting Information). We then trained an independent VAE using the 400‐ns REMD trajectory to explore conformations for the construction of our subsequent condensation simulation. This integration of REMD simulations and machine learning paves the way for effective conformational mining methodologies in future work.

Another challenge in the computational study of LLPS lies in its involvement of a large number of protein chains.^[^ ^39^, ^47^ ^]^ The phase separation of αSyn has been previously investigated using coarse‐grained force fields of varying resolution, including the multiple‐bead‐per‐residue MARTINI 3 force field^[^ ^53^ ^]^ and the one‐bead‐per‐residue HPS force field.^[^ ^13b^ ^]^ Similar coarse‐grained approaches have also been applied to other IDPs such as FUS and Tau.^[^ ^54^ ^]^ While these studies have provided valuable insights into the phase separation of IDPs, their limited resolution prevents the explicitly capture of the atomistic description of proteins, secondary structure transitions, and detailed physical interactions.^[^ ^39^, ^55^ ^]^ All‐atom simulations, while offering atomistic insights, are resource‐intensive when applied to the simultaneous simulation of multiple protein chains. In our research, we adopted a multiscale strategy to effectively study the spontaneous condensation of αSyn as well as the phase behavior and pre‐solidification of αSyn condensates. We utilized a cutting‐edge 3D computing cluster to conduct a microsecond‐scale, million‐atom all‐atom simulation to simulate the self‐association of 60 full‐length αSyn chains with their initial structures taken from an REMD monomer simulation combined with VAE networks. Our simulation marks the first instance of directly observing the spontaneous condensation and the pre‐solidification of the condensates for IDPs with such long sequences as αSyn at the level of atomistic resolution. It is noted that, due to the limited timescales accessible by current computational simulations, our condensation simulation captures only the very early stages of αSyn aggregation. This is evidenced by the continued increase in key physical parameters, such as β‐sheet probability and the number of H‐bonds, observed at the end of the simulation. We therefore conducted further coarse‐grained phase‐coexistence simulations that started from a preformed dense phase, incorporating secondary structure restraints from an all‐atom simulation. These simulations provide valuable insights into the thermoresponsive phase behavior of αSyn and explore the impacts of selectively removing β‐rich motifs on its phase separation properties.

The most important finding of this work is the identification of the β6 and β7 motifs as critical drivers of phase separation for αSyn, which has promising therapeutic implications. While many current treatments for neurodegenerative diseases focus on targeting pathological fibrils, increasing attention is being directed toward biomolecular condensates formed through phase separation as novel therapeutic targets.^[^ ^56^ ^]^ The important role of β6 and β7 in LLPS suggests that they may serve as potential targets for drug molecules that regulate the phase separation and maturation of αSyn. As a preliminary test, we examined the binding preferences of claramine^[^ ^57^ ^]^ and Lasioglossin III (LL‐III)^[^ ^58^ ^]^ on αSyn. These molecules have been previously reported to promote αSyn LLPS while inhibiting the conversion of droplets into fibrils. Molecular docking revealed that claramine and LL‐III bind to the β6 and β7 motifs in respectively 4 and 5 out of 9 docking runs (Figures S36 and S37, Supporting Information), suggesting that these motifs may play important roles in the regulatory mechanisms of these molecules. Recently, the Huang group combined advanced structural prediction tools with in silico virtual screening to design LLPS‐inhibitory peptides.^[^ ^59^ ^]^ A similar strategy could be employed for αSyn, whereby small‐molecule inhibitors targeting the β6 and β7 motifs may provide a compelling approach to modulate αSyn phase separation and prevent subsequent aggregation.

## 7 Conclusion

**Figure 7:**
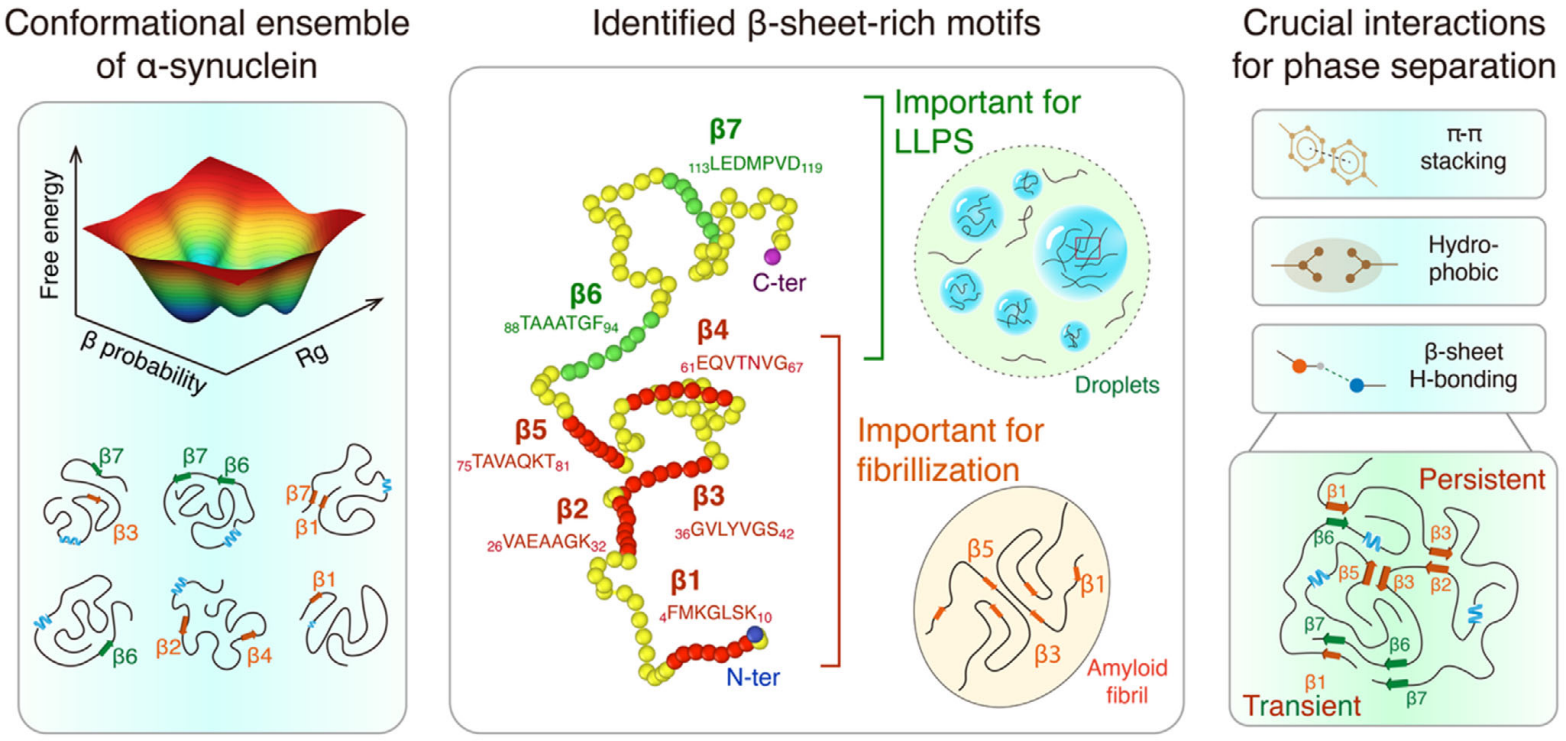
Schematic figure showing the identified seven β‐sheet‐rich motifs of αSyn and the phase separation mechanism revealed in this study. Left: Diagram of the conformational ensemble of monomeric αSyn, highlighting the multiple shallow free energy basins separated by low energy barriers and the conformational diversity. Middle: Diagram of the αSyn amino acid sequence, with key motifs for fibrillization and those for LLPS respectively, highlighted in red and green. Right: Depiction of the intermolecular interactions driving the phase separation of αSyn and the persistent and transient interactions between β‐sheet‐rich motifs.

In our study, we utilized a combination of multiscale simulations and experimental approaches to elucidate the molecular determinants that drive the phase separation of αSyn and the pre‐solidification of its condensates. Through REMD simulation on monomer and million‐atom MD simulation on condensation and pre‐solidification processes, we demonstrated the pivotal roles of β‐sheet H‐bonding, hydrophobic, and *π–π* interactions in driving phase separation. We identified seven β‐sheet‐rich motifs that form an interconnected network via persistent and transient H‐bonding interactions. Among them, β1‐β5 motifs provide persistent interactions that promote αSyn aggregation and are crucial for fibrillization, and β6/β7 motifs engage in transient H‐bonds that regulate the liquidity of the condensate and are critical for LLPS. Our predictions were validated by coarse‐grained simulations as well as ThT and imaging experiments on motif‐deleted αSyn variants. **Figure** **7** presents a schematic diagram depicting the multi‐basin conformational ensemble of monomeric αSyn, alongside the seven identified β‐sheet‐rich motifs and the phase separation mechanism elucidated in this study.

Collectively, this study identifies key residues essential for αSyn LLPS and fibrillization, reveals a close correlation between monomeric conformational properties and phase behavior of αSyn, and elucidates the molecular interactions driving αSyn LLPS. Our findings offer a comprehensive understanding of the molecular determinants governing αSyn phase separation. This research could lead to significant advances in treating diseases associated with αSyn by targeting the critical regions involved in LLPS and fibrillization.

## Conflict of Interest

The authors declare no conflict of interest.

## Supporting information



Supporting Information

Supplemental Movie 1

Supplemental Movie 2

Supplemental Movie 3

Supplemental Movie 4

## Data Availability

The data that support the findings of this study are available from the corresponding author upon reasonable request.
