# Supplementary material for: Dissecting the Molecular Determinants of α‐synuclein Phase Separation and Condensate Aging: The Pivotal Role of β‐Sheet‐Rich Motifs
Source: Adv Sci (Weinh). 2025 Sep 8;12(44):e11545. doi: 10.1002/advs.202511545 (PMC12667540; doi:10.1002/advs.202511545)
Supplement: Supplementary file 1 — Supporting Information [file ADVS-12-e11545-s001.pdf]

Dissecting the Molecular Determinants of α-synuclein Phase Separation and Condensate Aging: The Pivotal Role of β-sheet-rich Motifs

Supporting Information

Yiming Tang,[†] Jitao Wen,[†] Zhongyuan Yang,[†] Yifei Yao, Shaoshuai He, Jiyuan Zeng, Xuewei Dong, Qin Qiao, Yun Zhou, Sarah Perrett, Si Wu,* Guanghong Wei*

Yiming Tang, Zhongyuan Yang, Yifei Yao, Jiyuan Zeng, Yun Zhou, Guanghong Wei
Department of Physics, State Key Laboratory of Surface Physics, and Key Laboratory for Computational Physical Science (Ministry of Education), Fudan University, 2005 Songhu Road, Yangpu District, Shanghai 200433, China. E-mail: ghwei@fudan.edu.cn

Jitao Wen, Shaoshuai He, Si Wu, Sarah Perrett
Key Laboratory of Biomacromolecules, CAS Center for Excellence in Biomacromolecules, Institute of Biophysics, Chinese Academy of Sciences. 15 Datun Road, Chaoyang District, Beijing 100101, China. E-mail: wusi@ibp.ac.cn

Jitao Wen, Shaoshuai He, Si Wu
University of the Chinese Academy of Sciences, 19A Yuquan Road, Shijingshan District, Beijing 100049, China.

Xuewei Dong
Center for Soft Condensed Matter Physics and Interdisciplinary Research & School of Physical Science and Technology, Soochow University, 333 Ganjiang Road, Gusu District, Suzhou 215006, China.

Qin Qiao
Digital Medical Research Center, School of Basic Medical Sciences, Fudan University, 130 Dongan Road, Xuhui District, Shanghai 200032, China.

Sarah Perrett
Yusuf Hamied Department of Chemistry, University of Cambridge, Lensfield Road, Cambridge CB2 1EW, UK.

This PDF file includes:

Methods section
Supplementary text 1 and 2
Figures S1 to S37
Tables S1 to S6
Movie captions S1 to S4
Supplementary references

Other Supplementary Materials for this manuscript include the following:

Movies S1 to S4

Methods Section

All-atom replica-exchange molecular dynamics simulation of α-synuclein monomer

All-atom explicit-solvent replica-exchange molecular dynamics (REMD) simulation on α-synuclein (αSyn) monomer was performed using the GROMACS 2018.3 package [1] in combination with the Amber99SB-ILDN force field [2] (Table S1). The simulation contains 60 replicas with temperatures ranging from 308 to 410 K (Table S2) and a simulation time of 400 ns for each replica. The temperature range was selected to balance efficient conformational sampling, the preservation of biologically relevant structures, and computational cost. [3] The lowest temperature (308 K) ensures coverage of physiological conditions (i.e., 310 K), while the highest temperature (410 K) allows the system to overcome energy barriers efficiently. The temperature spacing between neighboring replicas was determined by solving the Metropolis criterion estimation of expected acceptance probability [4] with an optimized exchange probability of 20%. Similar temperature range and exchange probability has been used in a number of REMD studies on the study of folding and self-assembly of proteins/peptides [5]. To the best of our knowledge, this is the longest REMD simulation in explicit water for such a large protein consisting of 140 residues. The initial state of each replica was distinct from the others, with the initial conformation of αSyn monomers randomly selected from a 30-μs molecular dynamics (MD) simulation trajectory provided by D.E. Shaw Research. [6] The N-terminus was acetylated to mimic the physiological state of αSyn in cells. [7] Counter ions were added to neutralize the system and generate a salt concentration of 0.01 M. The particle mesh Ewald (PME) method [8] was utilized to calculate electrostatic interactions with a real space cutoff of 1.2 nm. The cutoff for van der Waals interactions was also 1.2 nm. The system was coupled to a temperature bath using a velocity rescale method and a pressure bath of 1 bar using the Parrinello-Rahman method. [9] The time constants for temperature coupling and pressure coupling were 0.1 ps and 2.0 ps, respectively.

Variational autoencoder for expanding the REMD-sampled conformational space

To further expand the conformational space sampled by REMD, we constructed a variational autoencoder (VAE) consisting of an encoder with five residual blocks and a structurally symmetric decoder. Each residual block integrates a batch-normalized fully connected layer with a ReLU activation function. The model comprises 115,397,064 optimizable parameters. The loss function was a weighted sum of (1) mean squared error (MSE) between the reconstructed and original αSyn conformations, and (2) a Kullback–Leibler divergence (KLD) term, ensuring that the latent space approximately follows a multivariate Gaussian distribution. At the initial stage of training, an additional MSE loss term was introduced to bias the backbone coordinates by enforcing similarity between the reconstructed and original αSyn backbone conformations. We trained the network separately using αSyn monomer conformations from the first 200 ns and 400 ns trajectories. To generate novel conformations beyond those captured in the REMD trajectory, we performed interpolation within the latent space projections of the simulation data.

Million-atom MD simulation on spontaneous condensation of αSyn

One of the major challenges in computational studies of LLPS is the involvement of a large number of protein molecules. Recent studies have employed coarse-grained force fields to investigate the phase behavior of various proteins. For example, Regy et al. examined the role of RNA in modulating the LLPS of the LAF1-RGG fragment using a system of 35 RNA chains and 70 LAF1-RGG chains. [10] Kapoor et al. investigated the phase separation of 50 HPα1 chains, [11] and Ingólfsson et al. studied the LLPS of full-length TDP-43 using 24 TDP-43 molecules. [12] In addition, all-atom simulations have also been employed but usually with smaller system size. For example, Galvanetto et al. explored the extreme dynamics within biomolecular condensates through all-atom simulations of 12 ProTα and 10 H1 molecules, [13] and Mukherjee simulated FUS condensates with a system of 8 FUS chains. [14] Inspired by these studies, we selected a system of 60 αSyn chains for our all-atom condensate simulation which is larger than previous all-atom studies and comparable in scale to prior coarse-grained simulations of protein LLPS.

The initial state of the condensation simulation includes 60 αSyn molecules with distinct conformations. To minimize bias from initial structures, we aimed to initialize the system with αSyn monomers exhibiting minimal ordered secondary structure. Specifically, we extracted conformations from the 310 K REMD trajectory at an interval of 1 ns and screened them for those containing fewer than 20 residues involved in β-sheets, β-bridges, or helices. A total of 52 conformations met this criterion. To further increase conformational diversity, we included 8 additional structures generated by the VAE model trained on the REMD data. These 60 structurally distinct αSyn monomers (Fig. S20) were then randomly placed into a 22 × 22 × 22 nm^3 simulation box, ensuring a minimum distance of at least 1.0 nm between any two chains. The simulation box was then solvated by 306,312 pre-equilibrated water molecules. Ions were added to neutralize the system and achieve a salt concentration of 0.01 M. The entire system was energy minimized using the steepest descent algorithm and equilibrated for 0.2 ns in the NVT ensemble and 200 ns in the NPT ensemble. The final state of this equilibrated system was used as the starting state of a 3.0 μs production run, which was conducted using the 3D computing software at the Xiaogan 3D Scientific Computing Center. The integration time step was set to 2 fs, and the trajectory was saved every 0.2 ns. The cutoff for van der Waals interactions and the real space cutoff for Coulomb interactions was set to 1.4 nm, which is slightly larger than that used in our REMD simulations due to the absence of a Verlet buffer in the condensate simulation. The system was coupled to an external temperature bath at 310 K and a pressure bath at 1 atm using the Nose-Hoover algorithm.

Coarse-grained phase coexistence simulations of αSyn_{WT}, $\alpha\text{Syn}_{\Delta\beta6}$, and $\alpha\text{Syn}_{\Delta\beta7}$

We conducted coarse-grained (CG) coexistence phase simulations for αSyn_{WT}, $\alpha\text{Syn}_{\Delta\beta6}$, and $\alpha\text{Syn}_{\Delta\beta7}$ using HOOMD-blue software in combination with the hydropathy scale (HPS) model. [10, 15] In addition to the bonded term connecting adjacent residues, angle terms were introduced for each set of three consecutive residues within the seven β-sheet-rich motifs. These angles were modeled using a harmonic potential

$U=1/2k(\theta-\theta_0)^2$, where the equilibrium angle θ_0 was universally set to 180°, and the spring constant k was assigned proportional to the β-propensity of the corresponding motif determined in our all-atom condensation simulation, with a proportional coefficient of 100. We generated the initial αSyn monomer conformations by random walk with all beads restrained within an 8×8×8 nm^3 cubic. We inserted 128 replicas of the afore-mentioned conformation into a 4×4×8 grid with a grid distance of 9 nm, maintaining a minimum distance between each two chains of at least 1 nm. The N-terminus of αSyn was uncharged to mimic the acetylated state, consistent with the all-atom simulation. The system was equilibrated for 500 ns in the NPT ensemble at a temperature of 150 K and a pressure of 1 bar. We then expanded the length of the z-dimension of the simulation box by a factor of ten to generate a slab-like box. The temperature was increased from 150 K to the target temperature for each simulation at a warming speed of 1 K/ns. Finally, the simulation was performed in the NVT ensemble for 4.0 μs. Periodic boundary conditions were applied in all simulations.

Analysis of simulation data

Data analyses were performed using our in-house codes and the tools implemented in the GROMACS, HOOMD-blue, MDAnalysis, and MDTraj packages. The last 350 ns data of the all-atom REMD trajectory, the last 2.0 μs of the million-atom condensation simulation, and the last 2.0 μs of each HPS simulation were used for statistical analysis. Helix and β-sheet probabilities were calculated using DSSP (version 3.0). The contact number for all-atom systems was defined as the number of atom pairs when the distance of two carbon atoms was ≤ 0.54 nm or the distance of any other atoms was ≤ 0.46 nm. The lifetime of the interaction between two residues was defined as the average time that these two residues continuously interact with each other. Inter-motif interactions with lifetimes shorter than the average lifetime across all motif pairs were classified as transient, whereas those exceeding the average were considered persistent. The correlation function was calculated by averaging over all autocorrelations of the existence functions (either 0 or 1) of all hydrogen bonds. Nonpolar contacts, which reflect hydrophobic interactions, were calculated using only aliphatic carbon atoms. When the distance between the hydrogen bond donor and acceptor was less than 0.35 ns, and the hydrogen-donor-acceptor angle was large than 150°, a hydrogen bond was considered to be formed. The cation-π interaction was considered to be formed when the distance between the centroid of aromatic rings and the centroid of the ε-amino group (NH_3^+) in the side chain of a Lys residue was ≤ 0.6 nm. The snapshot at each representative temperature was the central structure of the largest cluster. The critical temperature in the phase diagram was calculated by fitting it to the critical equation, $\rho_D-\rho_L=A(T_c-T)^\beta$. The parameter β was the critical scaling exponent (0.325, as in the university class of 3D-Ising model), and A was a protein-specific fitting parameter.

Protein expression and purification

The human full-length αSyn gene was subcloned into the pET-28a vector. The mutants were constructed by homologous recombination using the Hieff Clone® Plus One Step Cloning Kit. (Yeasen Biotechnology). The expression and purification of αSynWT and its variants, including $\alpha Syn_{\Delta\beta6\Delta\beta7}$, $\alpha Syn_{\Delta\beta6}$, $\alpha Syn_{\Delta\beta7}$, $\alpha Syn_{\Delta\beta3}$,

and $\alpha Syn_{\Delta\beta1}$, were carried out as previously described. [16] Briefly, the pET-28a plasmid carrying the αSyn gene was transformed into BL21 (DE3) competent cells. The single colony was then cultured in 2×YT medium at 37 °C under shaking at 220 rpm for 4 h. The cells were induced by adding 1 mM IPTG into the medium and cultured for 5 h. Cells were harvested and resuspended in a lysis buffer containing 100 mM Tris-HCl (pH 8.0), 300 mM NaCl, and 1 mM EDTA. The resuspended cells were boiled for 15 min and sonicated (Scientz-IIIE) on ice for 12 min with the pulse on for 5 s and off for 5 s. After centrifuging the cell lysate at 15000 rpm for 40 min, streptomycin sulfate powder was added to the supernatant to reach a final concentration of 20 mg/mL (w/v) with continuous stirring for 30 min in order to remove nucleic acid. The solution was then centrifuged at 15000 rpm for 15 min. The pH value of the supernatant was adjusted to 3.5 with 2 M HCl, followed by centrifugation at 15,000 rpm for 20 min. The supernatant containing αSyn protein was dialyzed to buffer A (20 mM Tris-HCl (pH 8.0), 1 mM EDTA) at 4 °C overnight. The solution was filtered with a 0.22-μm filter and loaded onto the Q column (GE Healthcare) at a speed of 2 mL/min, which was pre-equilibrated with Buffer A. The αSyn protein was then eluted using a 0–100% gradient of Buffer B (20 mM Tris-HCl (pH 8.0), 1 mM EDTA, and 1 M NaCl) over a duration of 40 min. The fractions containing αSyn protein were pooled, concentrated and further purified using a Superdex 75 column (GE Healthcare) with 30 mM Tris-HCl (pH 7.5) containing 100 mM NaCl as an elution buffer. The protein concentration was determined using a bicinchoninic acid assay (BCA) kit (Thermo Fisher Scientific). The protein was stored at −80 °C.

Detection of liquid-liquid phase separation of αSyn

αSyn was incubated at a concentration of 200 μM in 30 mM Tris-HCl buffer (pH 7.5) containing 10 mM NaCl in the presence of PEG 10,000 ranging from 4%-20%. The protein and PEG solutions were carefully mixed to avoid the formation of any bubbles. The turbidity assay was performed by adding 35 μL of the above αSyn protein solutions into a 384-well clear bottom plate. The absorbance at 400 nm was measured on a SpectraMax M3 plate reader (Molecular Devices) at 25 °C. At least three replicates were measured for each sample.

DIC and fluorescence imaging of phase-separated liquid droplets of αSyn were carried out on a laser scanning confocal microscope (Nikon A1, Nikon) equipped with a 100× oil-immersed objective (N.A.=1.4). The droplets were prepared by incubating 200 μM αSyn doped with 10 μM AF488-labeled αSyn in Tris-HCl buffer (pH 7.5) containing 10 mM NaCl and 18% PEG. A volume of 50 μL sample was added into a 96-well glass bottom plate (Cellvis). The samples were excited using a 488 nm laser.

Fluorescence recovery after photobleaching (FRAP)

FRAP experiments were performed on a laser scanning confocal microscope (Nikon A1, Nikon) equipped with a 100× objective (N.A.=1.4). The droplets formed by incubating 200 μM αSyn doped with 10 μM AF488-labeled αSyn were placed in a 96-well glass bottom plate (Cellvis). The laser for excitation and

bleaching was 488 nm. A region within the droplet was bleached by laser irradiation at 20 mW for 1 s. The time-lapse images after photobleaching were collected for 100 s at 1 frame per second. The fluorescence intensity within the bleached region was analyzed using Image J, and the proportion of the fluorescence recovery was calculated using the following equation:

$$\% \text{ Recovery} = [(Fi-F)/(F_0-F)] \times 100$$

where Fi is the fluorescence intensity after recovery of frame i, F is the fluorescence intensity immediately after photobleaching, and F_0 is the initial fluorescence intensity of the irradiation region before photobleaching.

ThT assay

The fibril formation of WT αSyn, $\alpha Syn_{\Delta\beta3}$, and $\alpha Syn_{\Delta\beta1}$ was measured by ThT assay. A concentration of 100 μM WT αSyn, $\alpha Syn_{\Delta\beta3}$ or $\alpha Syn_{\Delta\beta}$ was mixed with 20 μM ThT in 20 mM Tris-HCl buffer (pH 7.5) containing 10 mM NaCl. The samples were added into a 96-well plate (Costar) with 150 μL per well and incubated in a plate reader (BMG Labtech) at 37 ºC under orbital shaking at 600 rpm. The ThT fluorescence during amyloid formation was monitored every 30 min with excitation at 450 nm and emission at 485 nm. Three replicates were measured, and the fluorescence signal was averaged.

Supplementary Text 1

The reason for selecting Amber99SB-ILDN as all-atom force field that best describes α-synuclein.

Previous computational studies on αSyn have highlighted variations in results dependent on force fields. [6, 17] Although recent refinements in force field have improved the agreement of dimensions of IDPs with experimental estimates, they do not consistently predict the local secondary structure propensity. [18] In the case of monomeric αSyn, its Rg has been reported to vary widely in the literatures, [19] depending on the experimental technique and solution conditions used (Table S3). Although the Rg values observed in our REMD simulations are smaller than certain experimental estimates [19b] (Fig. S1A and Table S3), they are comparable to some experimental results. [19a] In addition, our simulation samples a wide distribution of Rg (Fig. S2) which overlaps with the experimentally measured range (Table S3). Despite some recently developed force fields providing larger Rg predictions than Amber99SB-ILDN (Fig. S1A), they fail to accurately predict secondary structures (Fig. S1B). Specifically, Amber99SB-ILDN-TIP4PD, Amber99SB-disp, and Amber03ws force fields predict considerably less β-sheet in αSyn (<7%) compared to results from CD (11.0%) and FTIR (15.6%) [20] (Fig. S1B), and Charmm36m force field assigns excessively high β-sheet probabilities (~80%) to a limited number of residues (Fig. S3). Considering the crucial role of β-sheets in fibril formation and phase separation of αSyn [21], we have chosen not to use the aforementioned force fields. Instead, we utilized the Amber99SB-ILDN force field with a well-established reputation in computational studies for αSyn. [22]

Supplementary Text 2

Comparison of residue-based β-sheet propensities predicted by simulations and those revealed by NMR studies.

Various papers in the literature have reported nuclear magnetic resonance (NMR) spectroscopy studies on full-length human αSyn and have deposited the corresponding chemical shift data to the biological magnetic resonance data bank (BMRB) database. We have selected three NMR studies [23] whose experimental conditions were, respectively, (1) in living cell at 277 K, (2) at 15 °C, pH 7.4, with 50 mM NaCl and 25 mM Hepes·NaCl buffer, and (3) at 20 °C, pH 6.5, with 200 mM NaCl and 20 mM Sodium Phosphate buffer. Although the conditions are slightly different, these experiments were all performed in cellular environment or *in vitro* at near neutral pH. We utilized the δ2D program [24] to transform the chemical shift data into β-sheet propensity predictions (Fig. S8). We observed notable discrepancies in secondary structure of αSyn predicted by different studies (Fig. S8). To quantify these differences, we calculated the backbone root-mean-square deviation (RMSD) between the β-sheet distribution profiles obtained from various NMR datasets. Similarly, we assessed the deviation between our REMD predictions and the experimental data by computing the backbone RMSD between the REMD-predicted β-sheet distribution and

those derived from the NMR studies. The RMSD values between different NMR datasets [23] range from 0.70 to 1.48, while our computational predictions show RMSD values of 1.02, 1.27, and 1.79 relative to the three NMR datasets (Fig. S9). These results indicate that the deviations between our REMD predictions and the experimental data are comparable to, and in some cases smaller than, the discrepancies among the NMR studies themselves. We then calculated the averaged residue-based β-sheet probability based on the three sets of chemical shift data (Fig. S10). While CD and FTIR suggest a β-sheet content over 10% for monomeric αSyn [20b] (Fig. S1B), NMR data indicates a much lower propensity (<6%, Fig. S10). This discrepancy likely arises from the transient nature of β-sheet formation in αSyn's disordered state. [20a, 25] Nevertheless, the majority of peaks identified in the NMR-predicted β-sheet distribution are also present in the REMD-predicted distribution (Fig. S10). To further quantify this observation, we identified β-sheet-peak residues in both distributions using a moving-window approach with a window size of five residues. Specifically, five consecutive residues were classified as β-sheet-peak residues if their average β-sheet probability exceed that of the five preceding and five succeeding residues. We found reasonable agreement between the β-sheet peaks predicted by NMR and those predicted by REMD, despite minor positional shifts (Fig. S11A). We also compared the seven β-sheet-rich motifs identified in our original manuscript with the NMR-predicted β-sheet peaks and found that all seven motifs overlapped with NMR-derived peak regions (Fig. S11B). These results demonstrate that our REMD simulations produce β-sheet distributions in agreement with experimental data and that the seven identified β-sheet-rich motifs are supported by existing NMR evidence.

Supplementary Figures

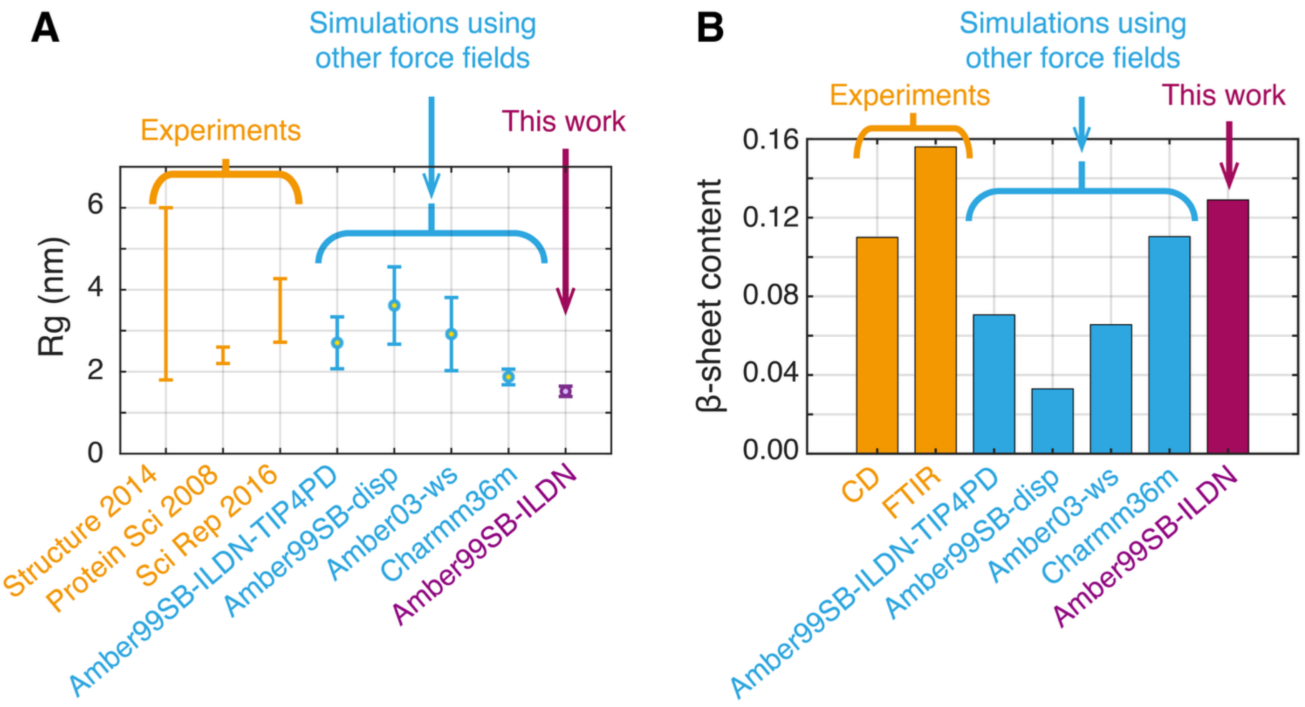

Fig. S1.

Radius of gyration (Rg) and β-sheet contents of monomeric αSyn from different studies. Displayed are three types of estimates with color-coded. Yellow: experimentally derived values, [19-20] b lue: predictions from MD simulations using recently-developed force fields, purple: results from our REMD simulation employing the Amber99SB-ILDN force field. Predictions from the newly developed force fields (blue bars) were calculated using trajectories provided by D.E. Shaw Research. [6]

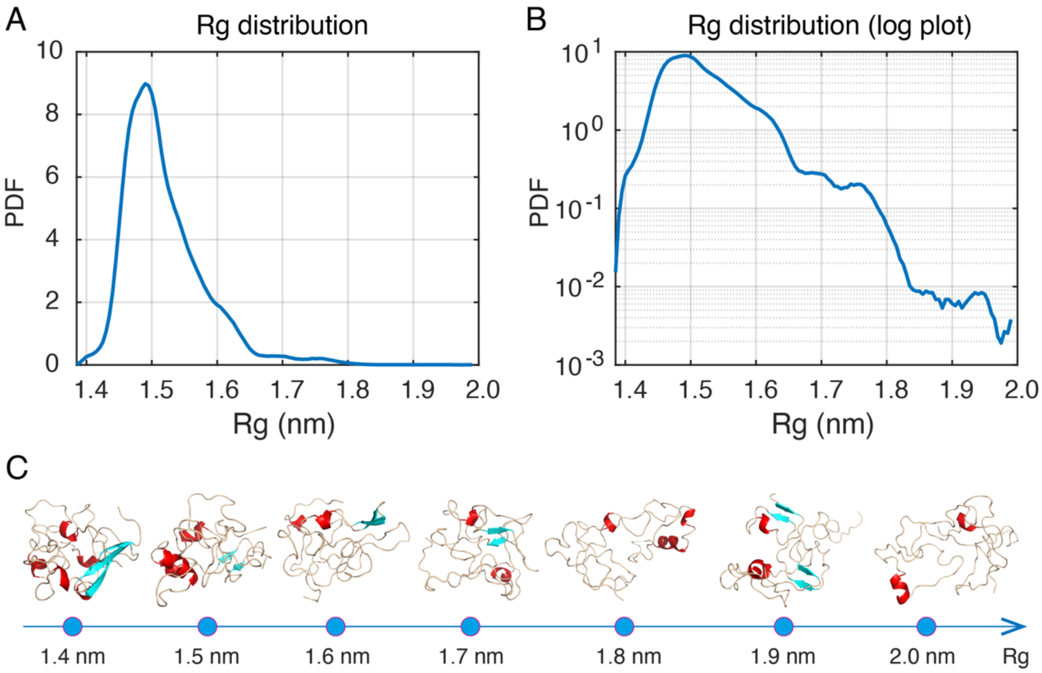

Fig. S2.

Rg distribution of the αSyn monomer at 310 K. (A, B) Probability density function (PDF) of Rg values of all conformations in the 310 K replica of the REMD simulation, shown in (A) linear scale and (B) logarithmic scale. (C) Representative conformations of the αSyn monomer with varying degrees of compaction, illustrating Rg values ranging from highly compact (~1.4 nm) to extended (~2.0 nm) states.

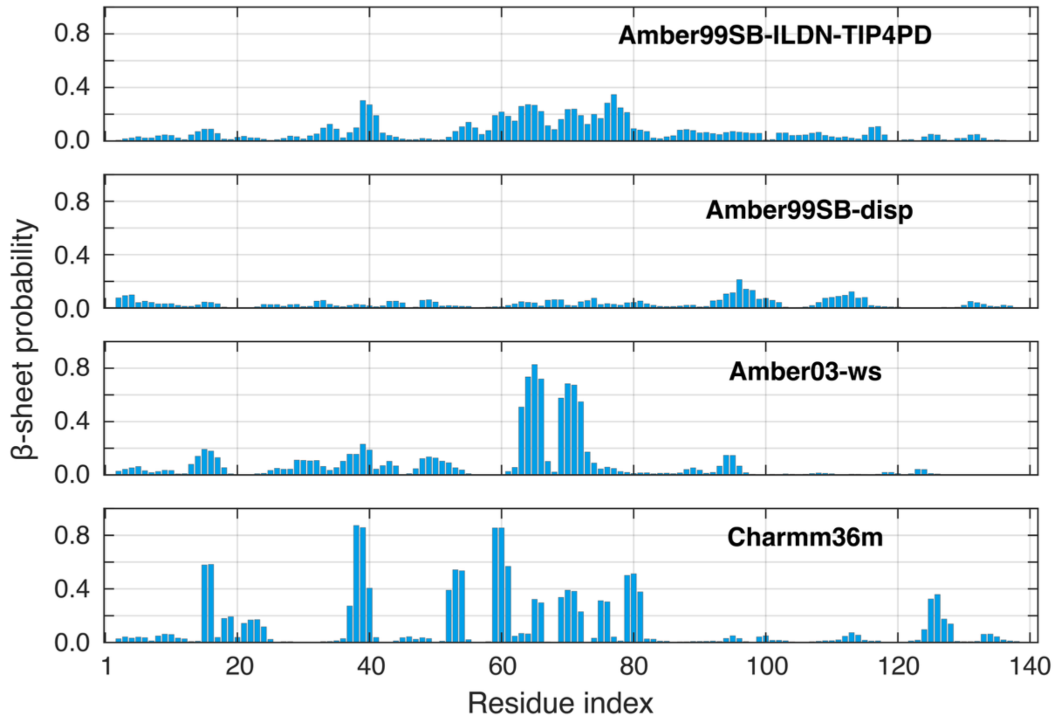

Fig. S3.

Residue-based β-sheet propensities predicted by MD simulations using four recently developed force fields. The results were calculated using trajectories provided by D.E. Shaw Research. [6]

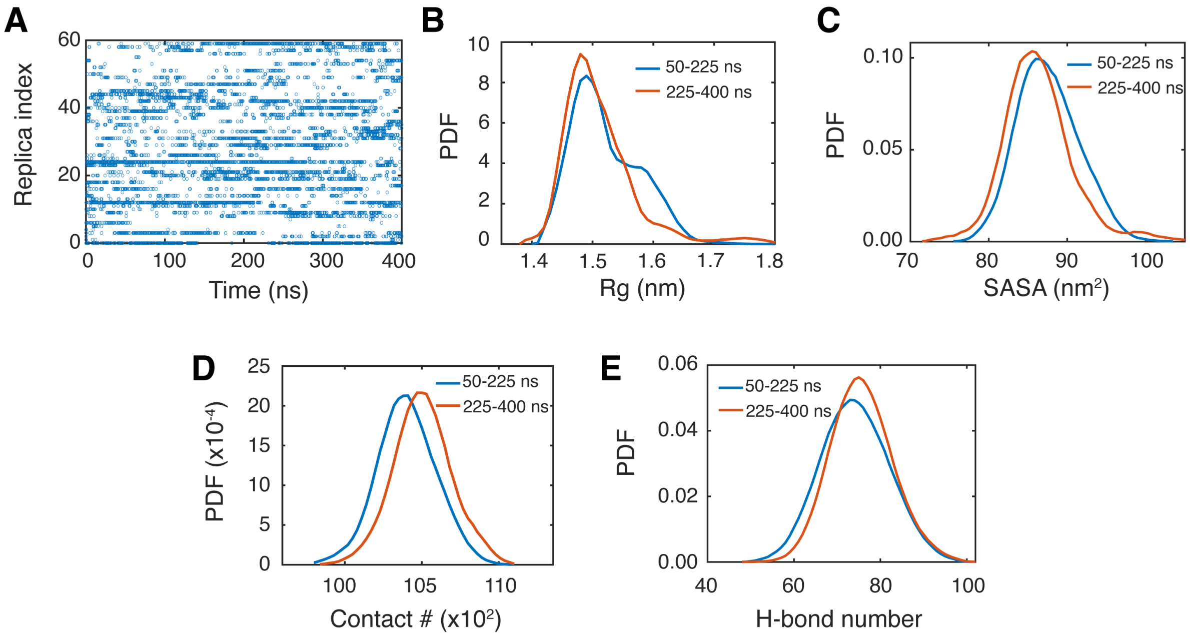

Fig. S4.

Convergence assessments for the REMD simulation of αSyn monomer. (A) Replica index composing the simulation trajectory at 310 K, plotted as a function of simulation time, showing that the 310 K trajectory comprises contributions from all 60 replicas. **(B-E)** Probability density function (PDF) for various structural parameters across two non-overlapping time windows (50-225 ns and 225-400 ns): **(B)** radii of gyration (Rg), **(C)** solvent-accessible surface area (SASA), **(D)** total contact number, and **(E)** hydrogen-bond (H-bond) number.

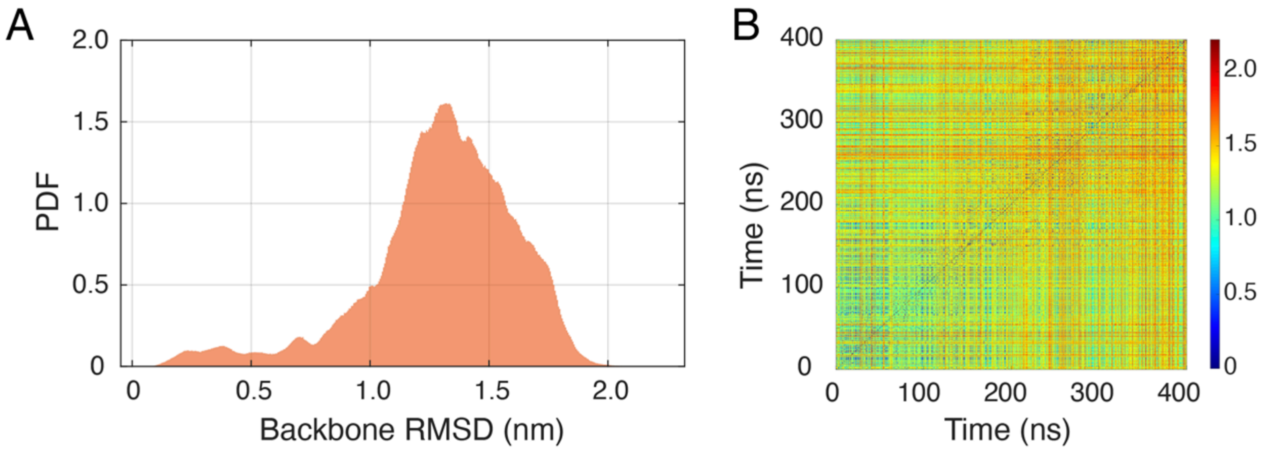

Fig. S5.
RMSD analyses of the conformations sampled by the 310-K replica of our REMD simulation. (A) Probability density function (PDF) of backbone RMSD values between all pairs of conformations sampled in the 310 K replica. **(B)** The RMSD matrix showing the RMSD values of each pair of conformations.

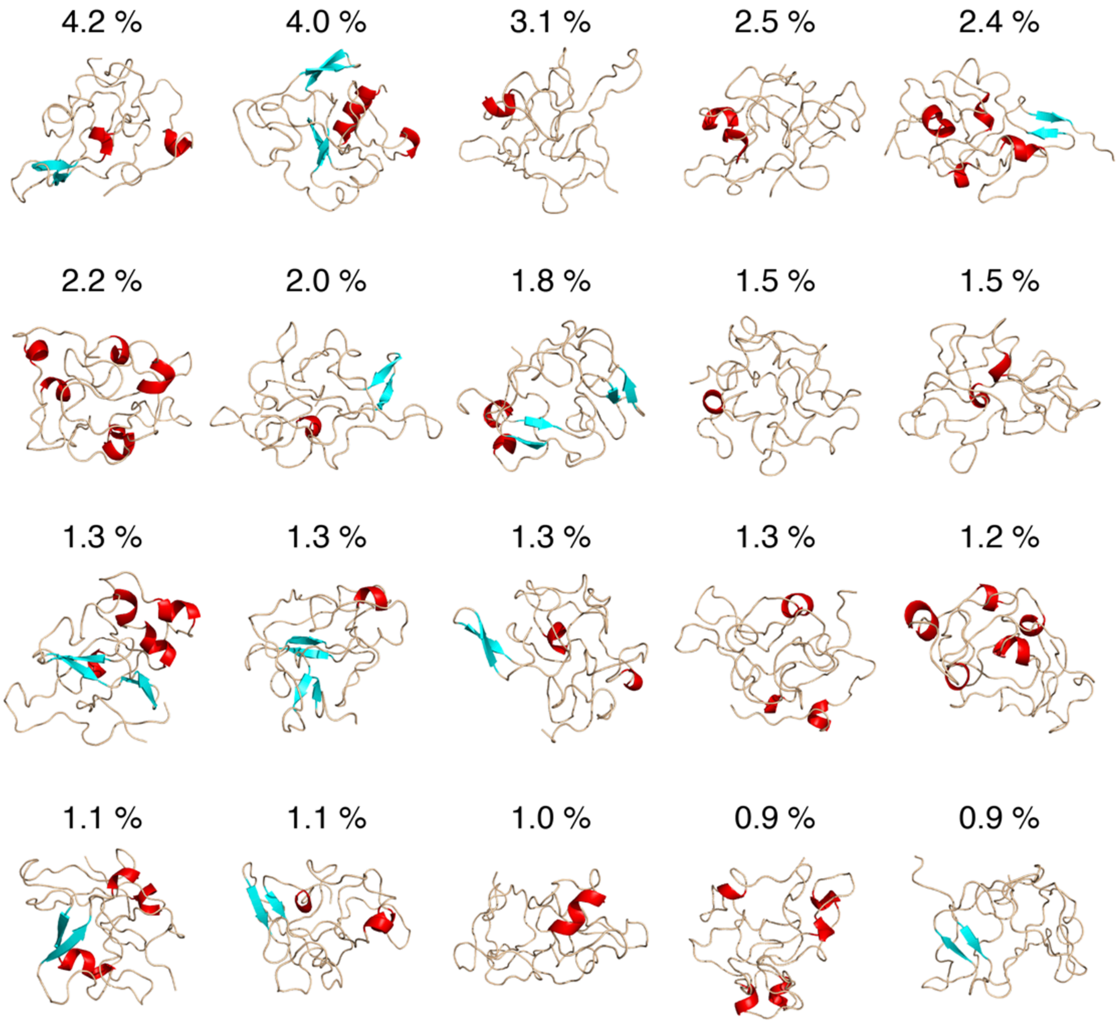

Fig. S6.

Central structures of the 20 largest clusters identified from the 310 K replica of our REMD simulation. The proportion of structures within each cluster is shown.

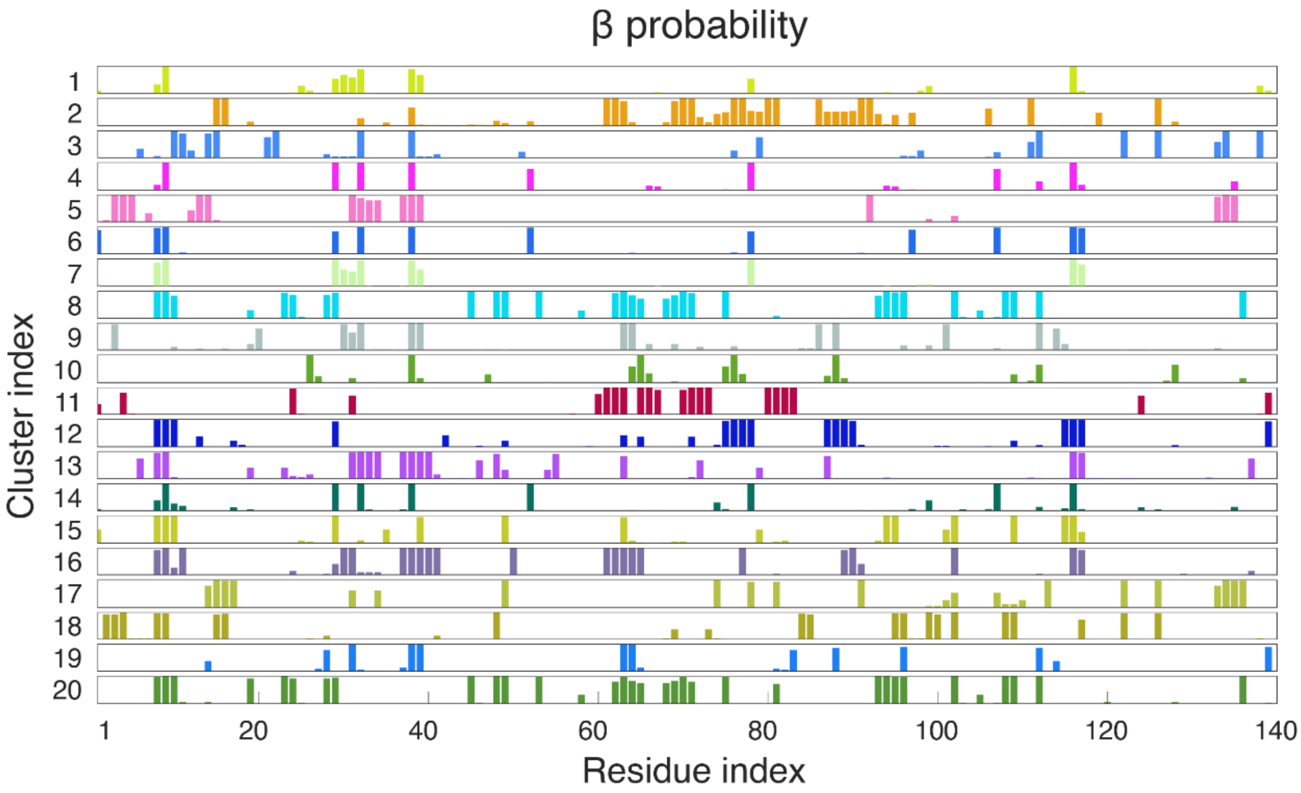

Fig. S7.
β propensities of all αSyn monomer conformations in each of the 20 largest clusters at the residue level.

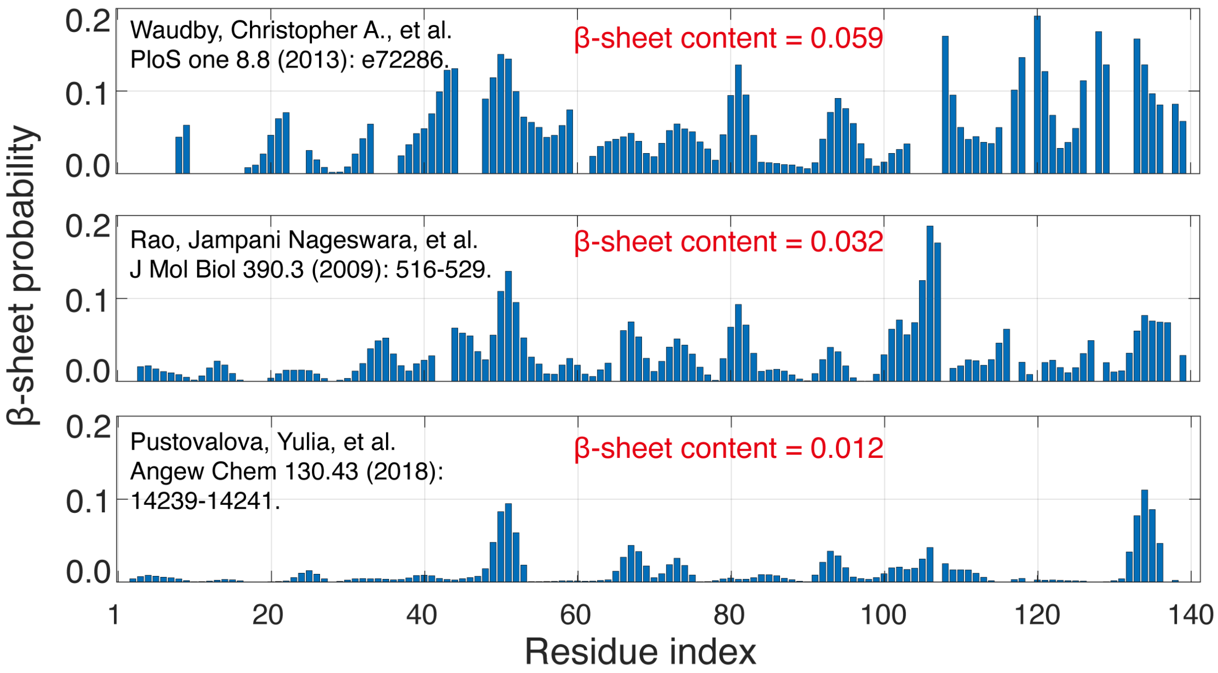

Fig. S8.

Residue-based β-sheet probabilities predicted by chemical shift data from three previous NMR studies. [23]

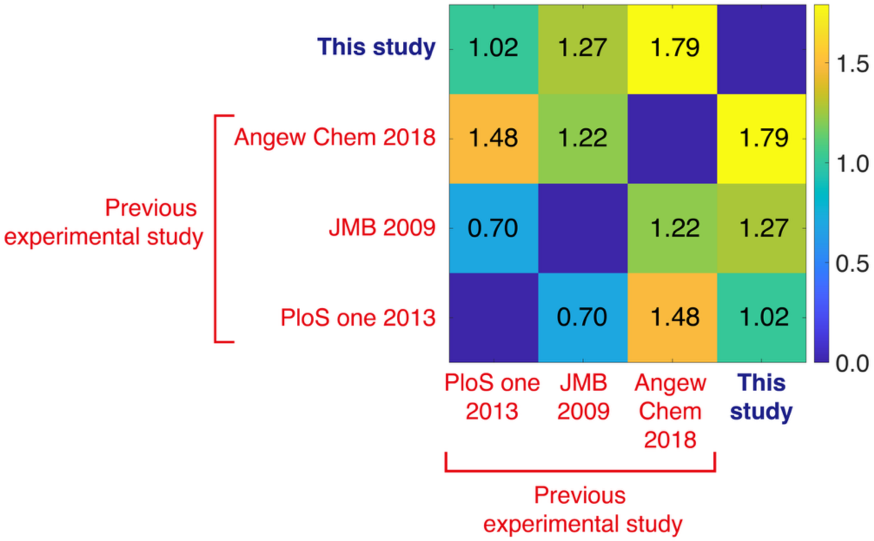

Fig. S9.

Root-mean-square deviation (RMSD) of β-sheet distributions along the αSyn sequence, calculated between each pair of NMR datasets [8-10] and between each NMR dataset and our REMD predictions. The β-sheet distribution was defined as the residue-wise β-sheet probability as a function of residue index. Residues lacking chemical shift data are excluded from the calculation. To ensure comparability, the β-sheet probabilities for each NMR dataset and the REMD prediction were normalized by their respective mean values.

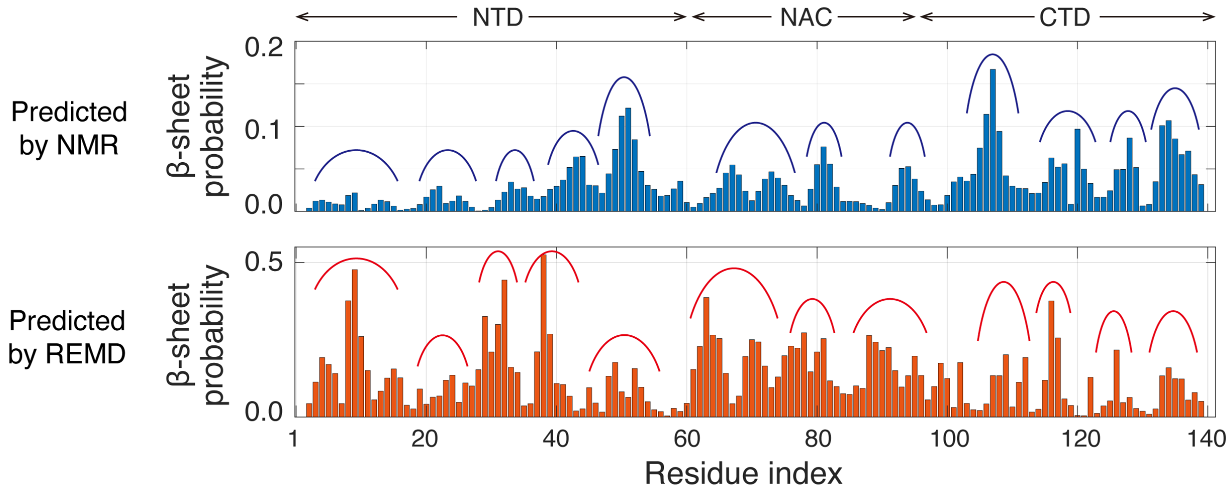

Fig. S10.

Residue-based β-sheet probabilities observed experimentally and those predicted by our REMD simulation. The experimental predictions were calculated by the δ2D program [24] designed for disordered protein. It takes the NMR-derived chemical shift data as input and predict the β-sheet probability for each residue. β-sheet probability profiles predicted from three previous NMR studies [23] were averaged.

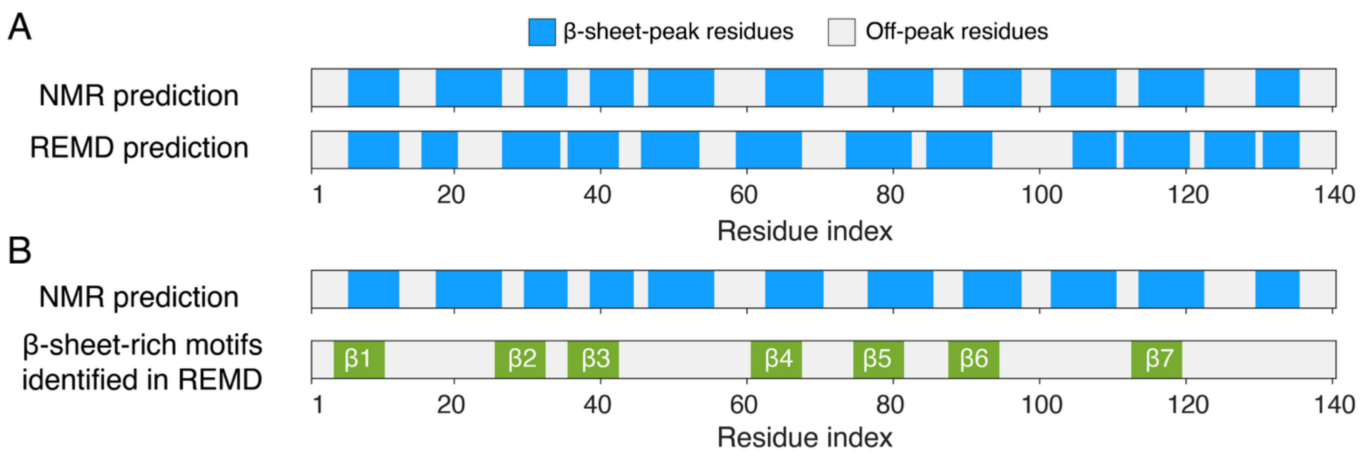

Fig. S11.

Comparison of NMR identified β-sheet peaks residues with computational predictions.

(A) Comparison of β-sheet peaks identified from NMR chemical shift data [8-10] and those predicted by our all-atom REMD simulations. Peaks were defined using a moving-window approach with a window size of five residues. (B) Comparison of β-sheet peaks identified from NMR data and the seven β-sheet-rich motifs identified in our REMD simulations.

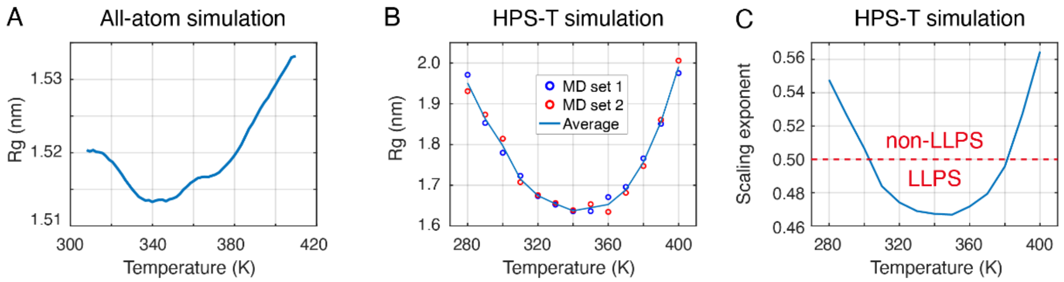

Fig. S12.

The temperature dependence of αSyn monomer Rg. Rg profile determined by **(A)** all-atom and **(B)** two independent sets of HPS-T simulations. **(C)** The temperature dependent of Flory scaling exponent determined by HPS-T simulations.

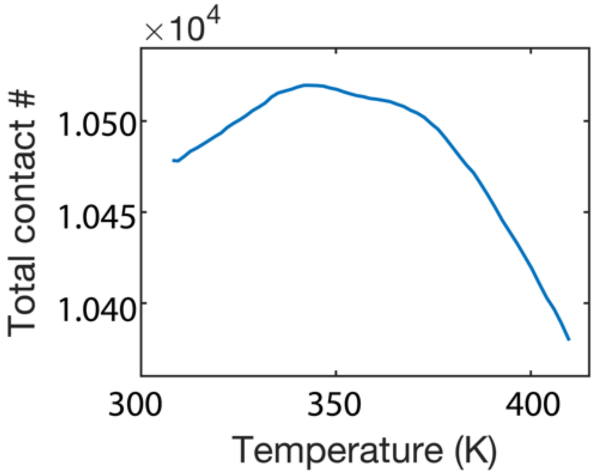

Fig. S13.
Contact numbers for intramolecular interactions within monomeric αSyn as a function of temperature.

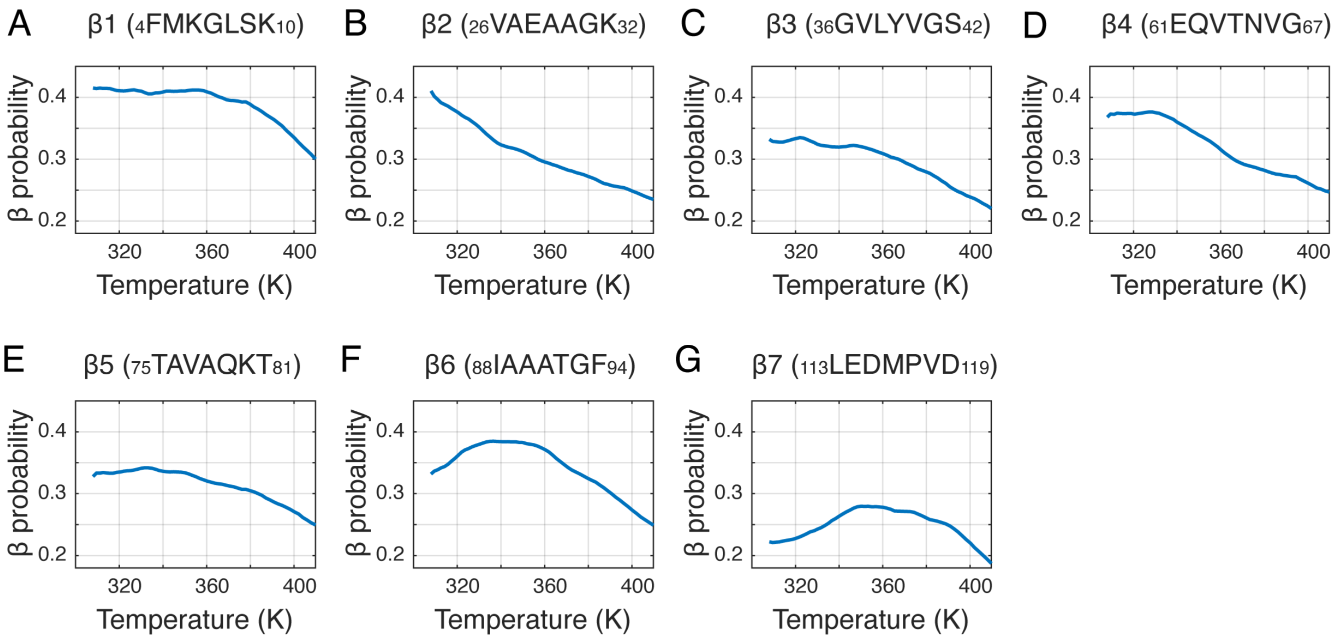

Fig. S14.
Temperature dependence of β probabilities of the seven β-sheet-rich motifs.

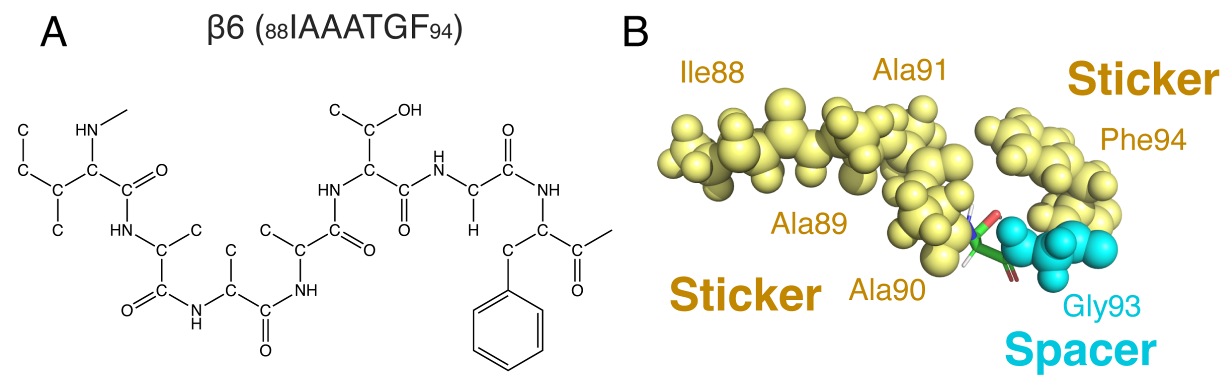

Fig. S15.
Sequence characteristics of the β6 motif. (A) Chemical structure of the β6 motif, with hydrogens bonded to carbon atoms omitted for clarity. **(B)** Schematic representation of the sticker-spacer characteristics of the β6 motif. The stickers ($_{88}$IAAA$_{91}$ and F94) are highlighted in yellow, while the spacer (G93) is shown in blue.

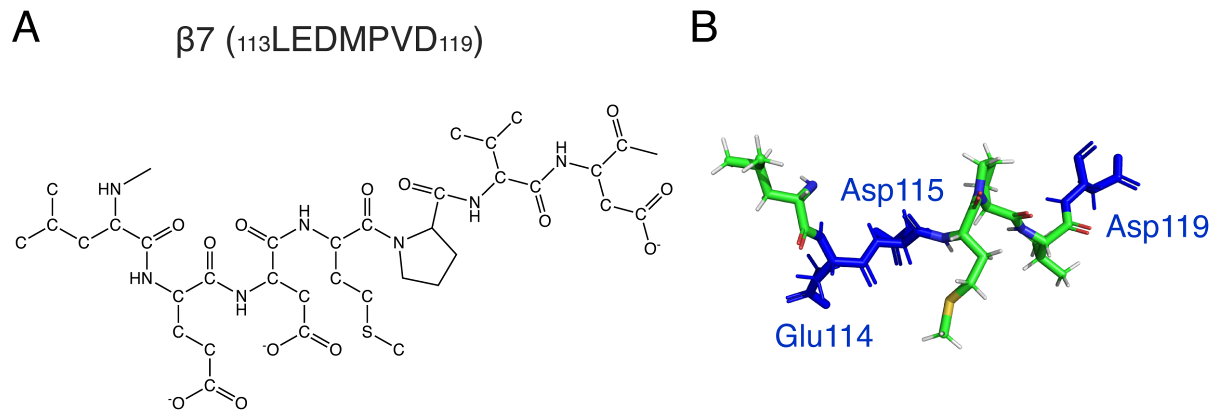

Fig. S16.
Sequence characteristics of the β7 motif. (A) Chemical structure of the β7 motif, with hydrogens bonded to carbon atoms omitted for clarity. **(B)** Stick representation highlighting the enrichment of negatively charged residues (blue color) in the β7 motif.

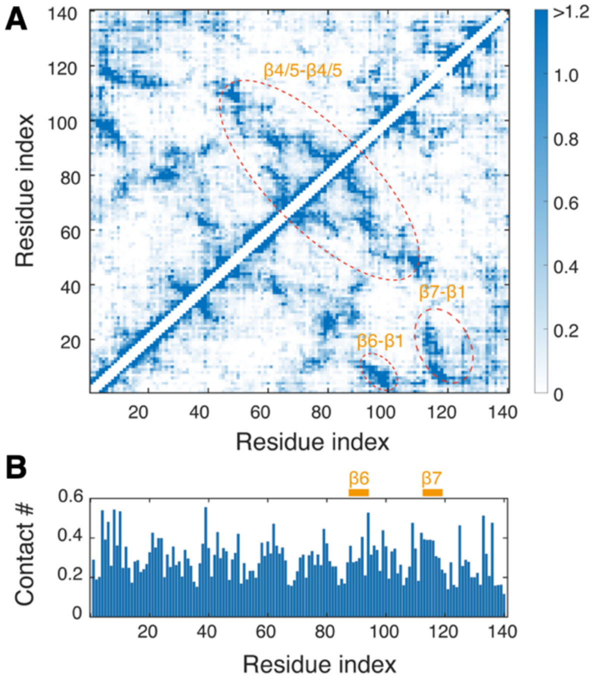

Fig. S17.

Intramolecular interactions analysis of αSyn at 310 K. (A) Intramolecular contact numbers between each pair of residues. **(B)** Cumulative contact numbers for each residue with all other residues.

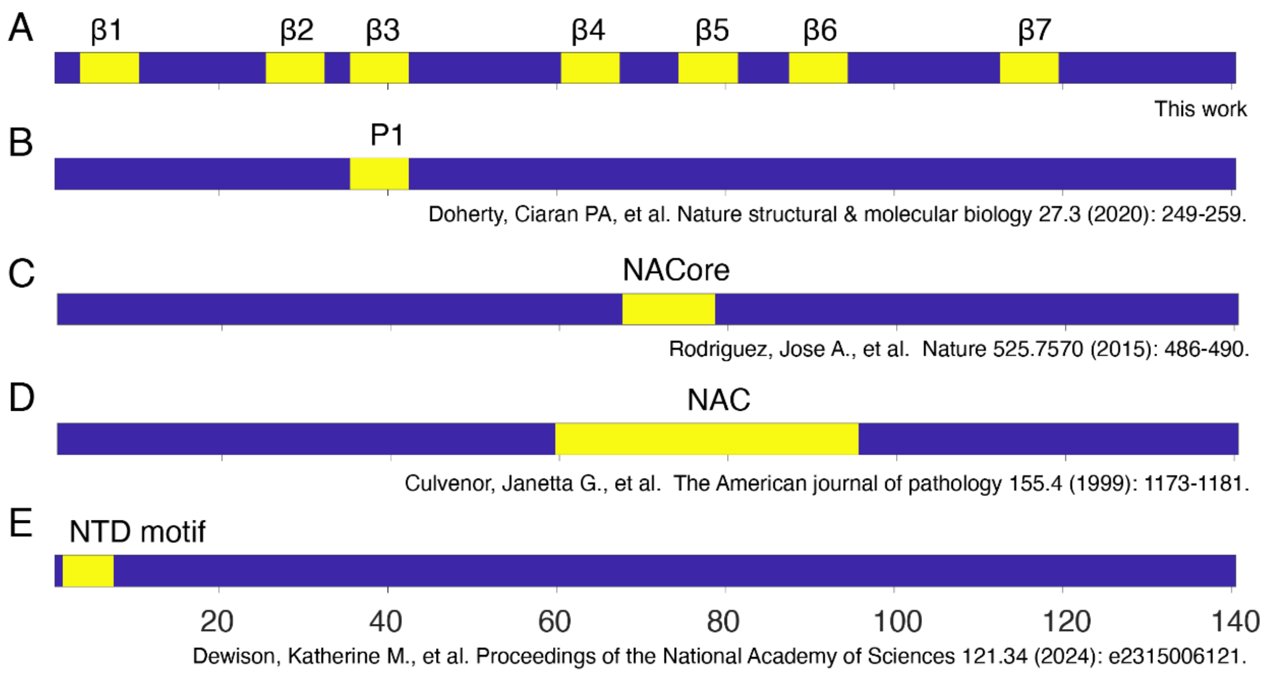

Fig. S18.

Illustration to the location of peak residues in β-sheet profiles. The location of (A) seven β-sheet-rich motifs and (B-E) experimentally-determined aggregation prone regions along the amino acid sequence of αSyn.

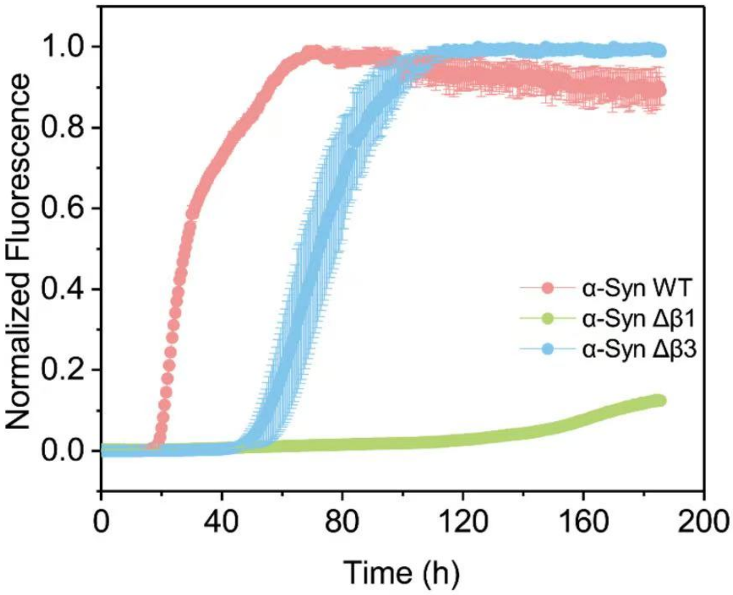

Fig. S19.
The fibrilization process of wildtype αSyn (αSyn$_{WT}$) and αSyn with β1 or β3 deleted (αSyn$_{\Delta\beta1}$ and αSyn$_{\Delta\beta3}$) measured by ThT assay.

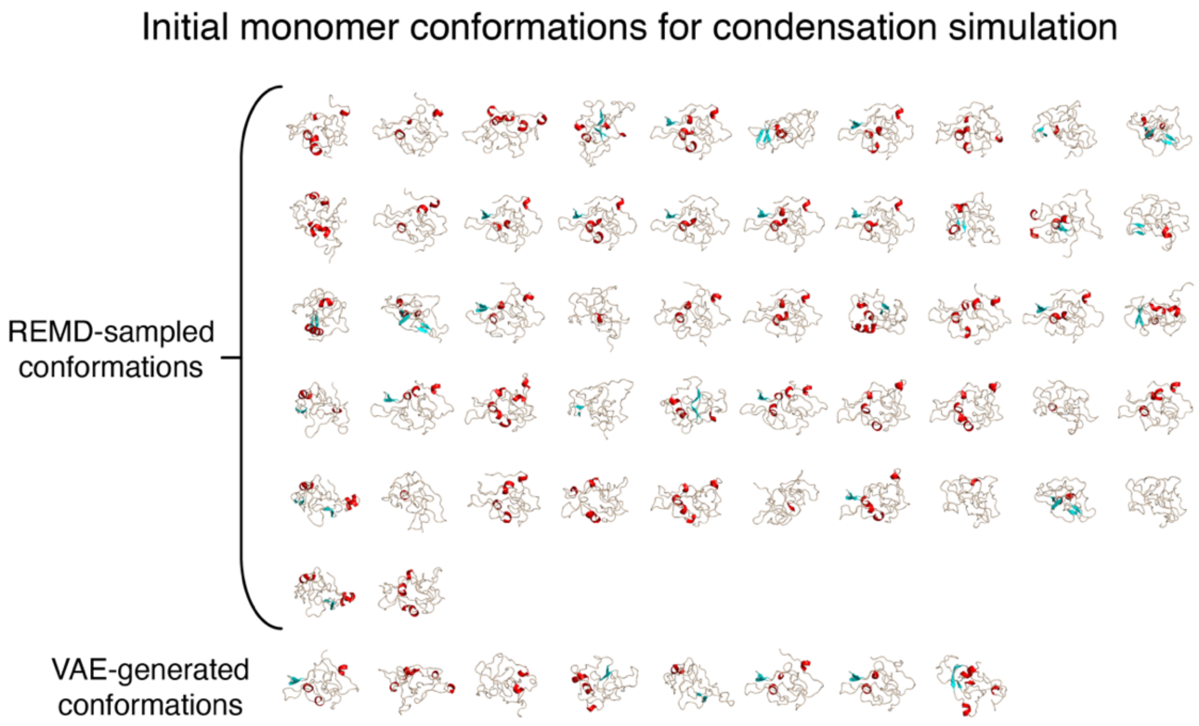

Fig. S20.
Initial monomer conformations used in the condensation simulations. Among them, 52 conformations were selected from the REMD simulation trajectory, while the others were generated using the VAE network.

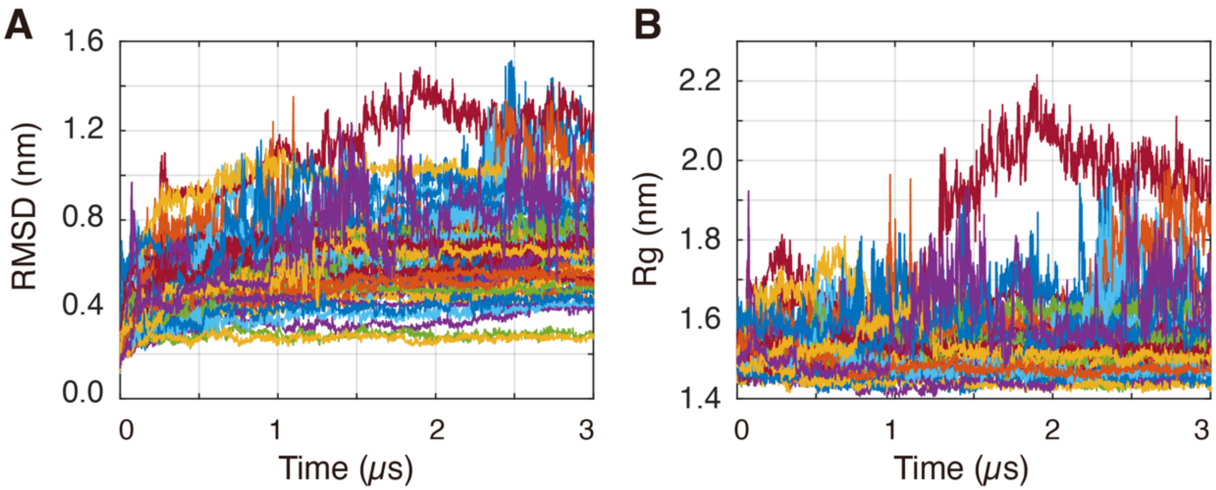

Fig. S21.
The time evolution of (A) backbone RMSD and (B) Rg of each of the 60 αSyn chains within the condensation simulation.

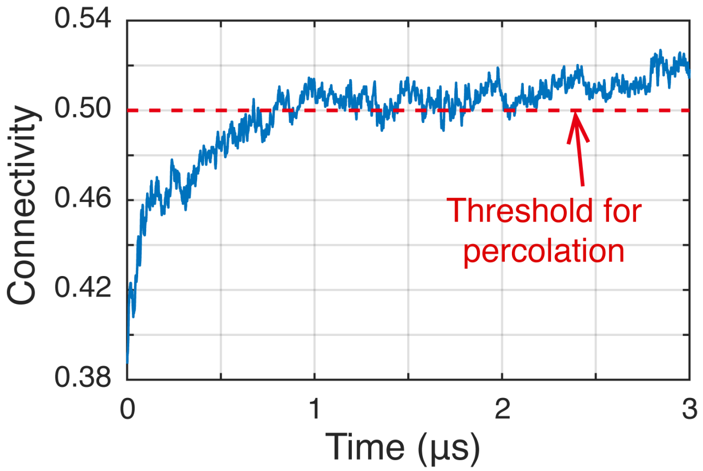

Fig. S22.
The network connectivity of all 60 αSyn molecules as a function of simulation time. The threshold for percolation (calculated using a cubic grid) is shown as the red dash line.

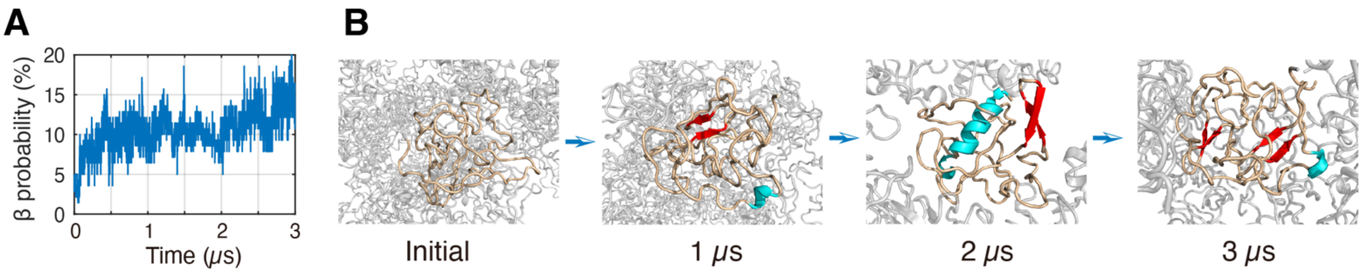

Fig. S23.

The formation and growth of β-sheet of a representative chain. (A) Evolution of β-sheet probability for a single chain. **(B)** Snapshots at four time points for a single chain. The β-sheets and helices for the representative chain are highlighted in red and green, respectively. Other chains are shown in gray cartoon representation.

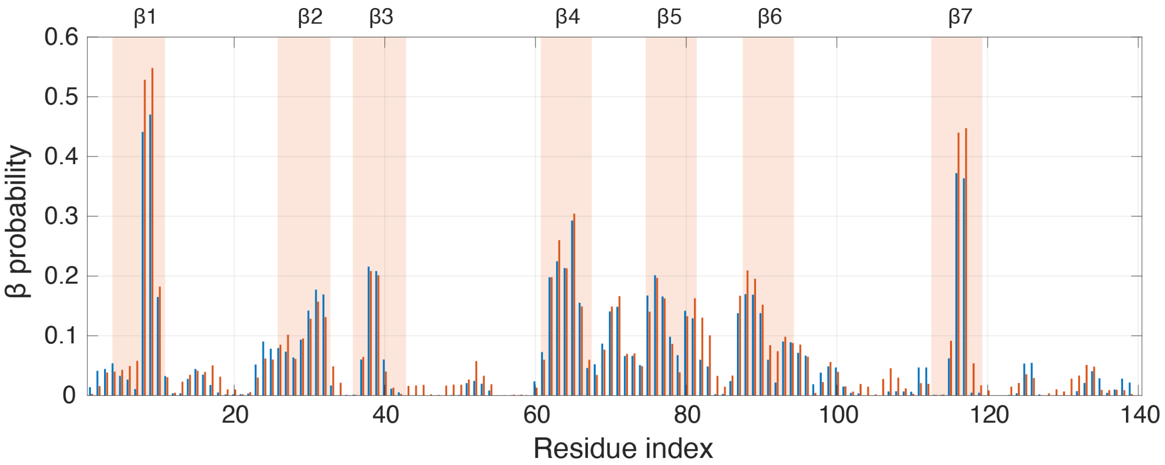

Fig. S24.

Residue-based β-sheet probability of αSyn in the condensation simulation. The data is averaged over 60 αSyn chains, across two time intervals (0-0.1 μs and 2.0-3.0 μs) of the condensation simulation. The β-sheet-rich motifs (identified by our monomer REMD simulation) are highlighted by orange shading.

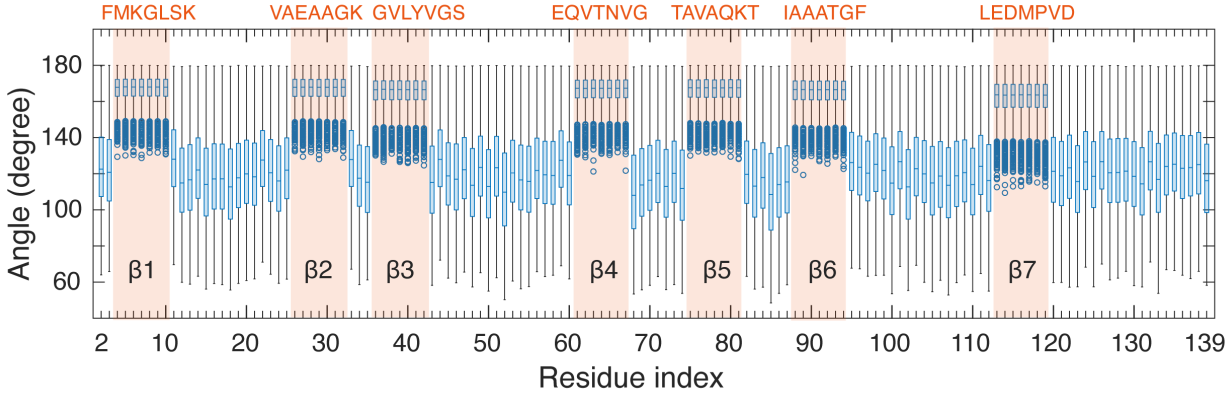

Fig. S25.

Box chart plot of the mainchain angles formed by each set of three adjacent residues. The seven β-sheet-rich motifs are shaded in orange.

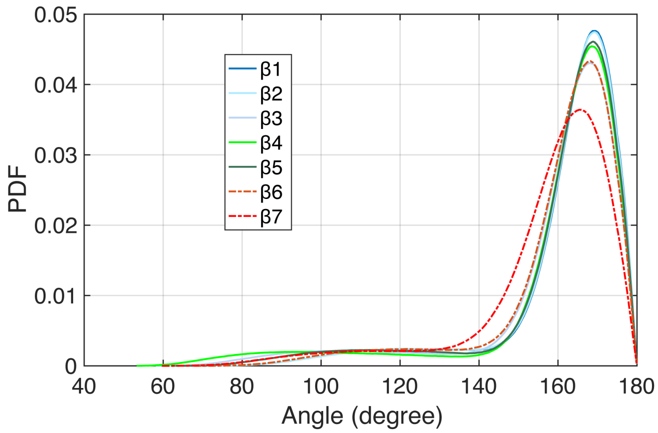

Fig. S26.

Distribution of the mainchain angles formed by each set of three adjacent residues within the seven β-sheet-rich motifs

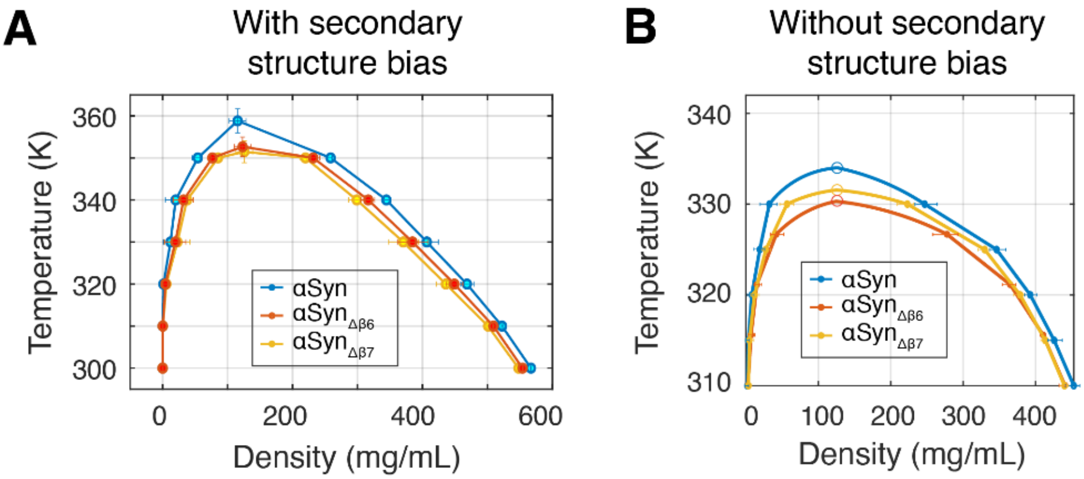

Fig. S27.

The phase diagrams of αSyn$_{WT}$, αSyn$_{\Delta\beta6}$, and αSyn$_{\Delta\beta7}$ as a function of density and temperature, obtained by simulations using **(A)** HPS force field with secondary structure constraints and **(B)** original HPS force field without secondary structure constraints.

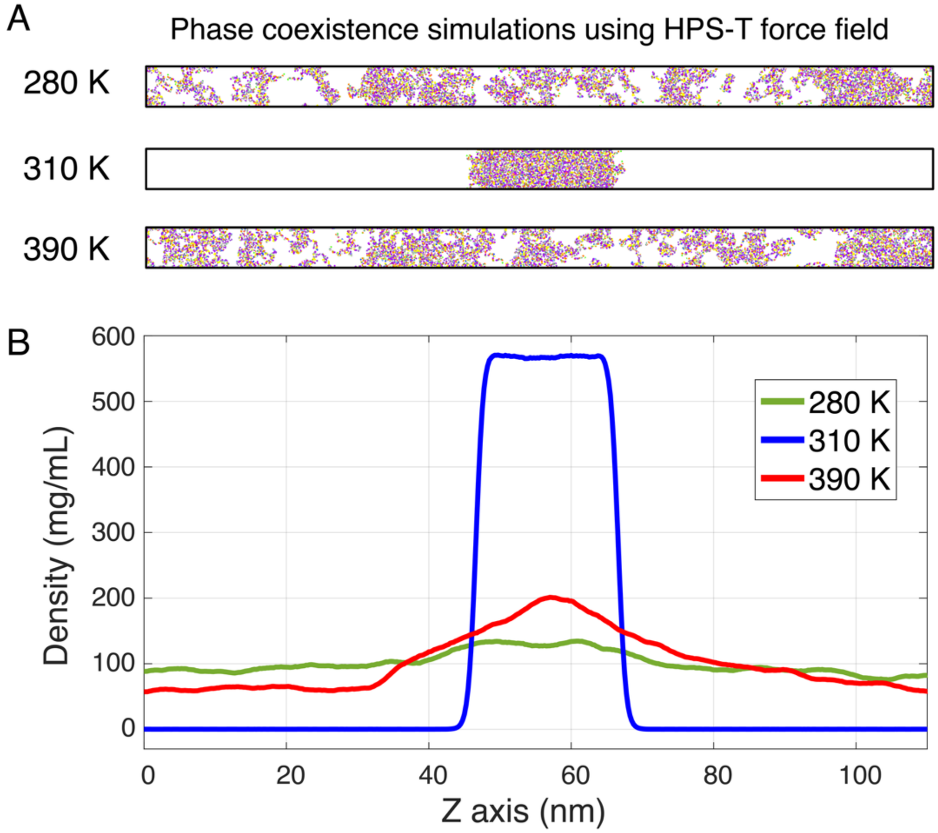

Fig. S28.

Coarse-grained phase coexistence simulations using the HPS-T force field. (A) Final snapshots to the phase coexistence simulations at three different temperatures. **(B)** Slab density profiles at three different temperatures.

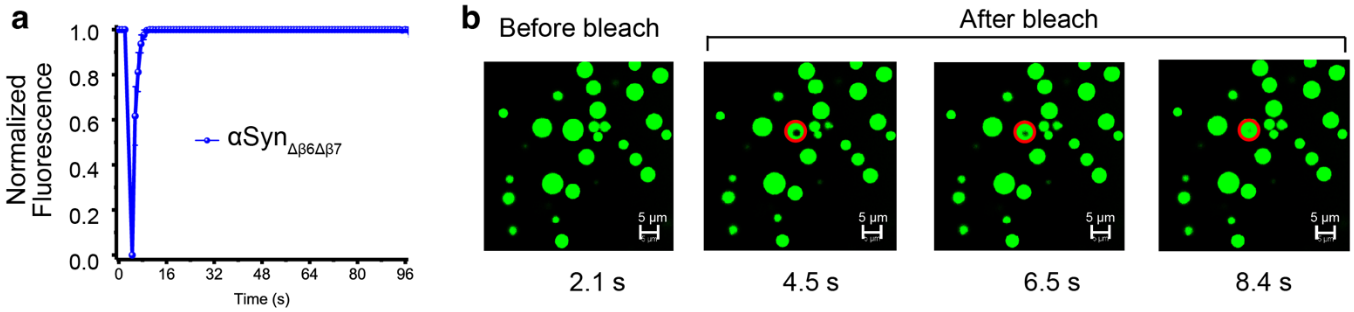

Fig. S29.

FRAP analysis of droplets formed by αSyn$_{Δβ6Δβ7}$. (A) The normalized fluorescence intensity, and **(B)** the fluorescence images of αSyn$_{Δβ6Δβ7}$ droplets during the fluorescence recovery process after photo bleaching.

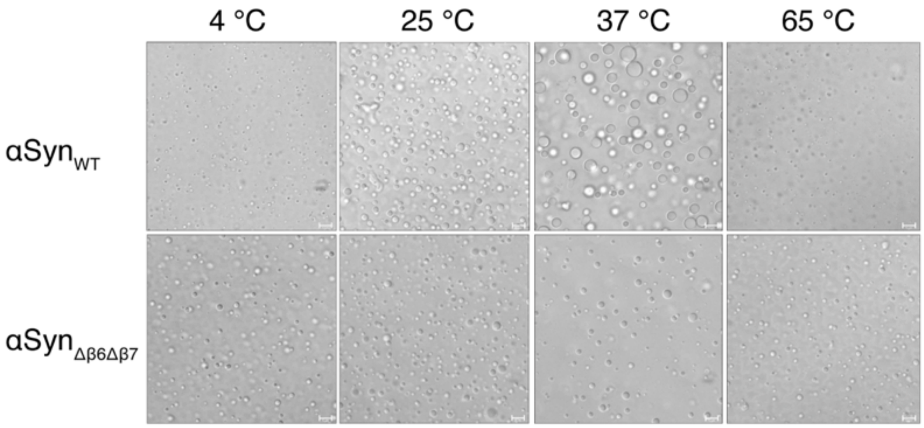

Fig. S30.

DIC images of the droplets formed by WT αSyn and αSyn$_{\Delta\beta6\Delta\beta7}$ at different temperatures as indicated.

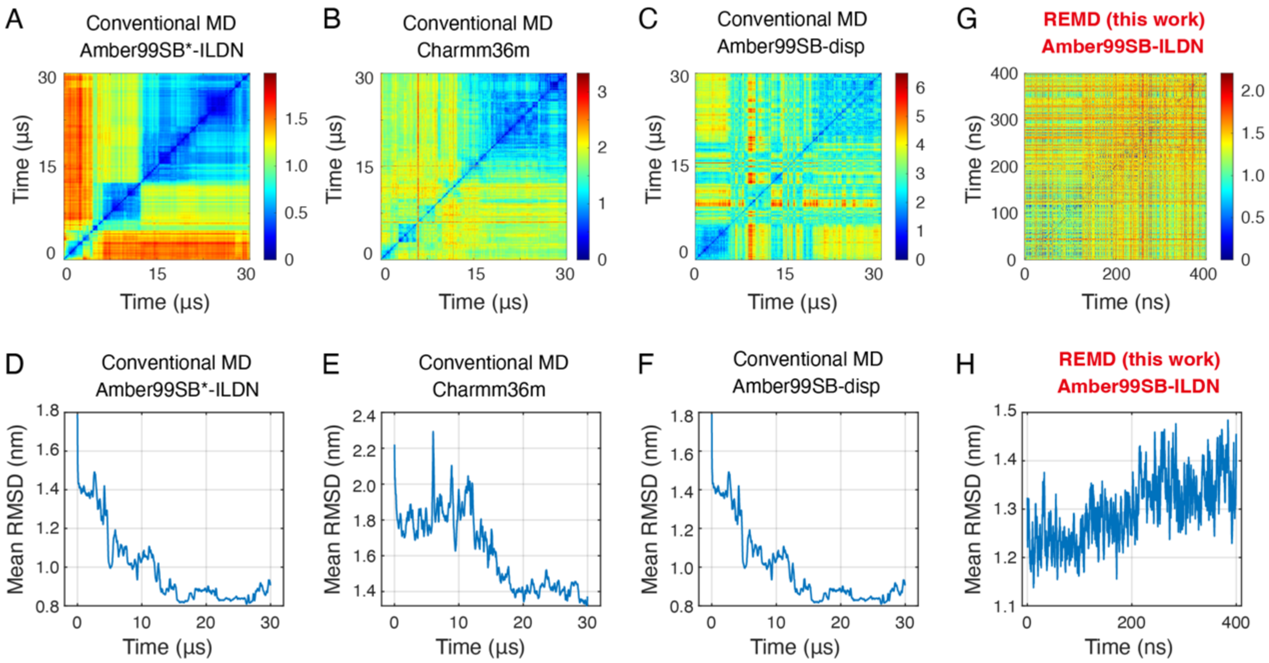

Fig. S31.

RMSD values between each pair of conformations within simulation trajectories. (A-C) The RMSD matrix showing the RMSD values of each pair of conformations sampled by 30-μs conventional MD by D.E. Shaw [7] using (A) Amber99SB*-ILDN, (B) Charmm36m, and (C) Amber99SB-disp force fields. **(D-F)** The average RMSD of each frame relative to all other frames for the three force fields. **(G-H)** The RMSD matrix and time evolution of average RMSD of our 400-ns REMD using Amber99SB-ILDN forcefield.

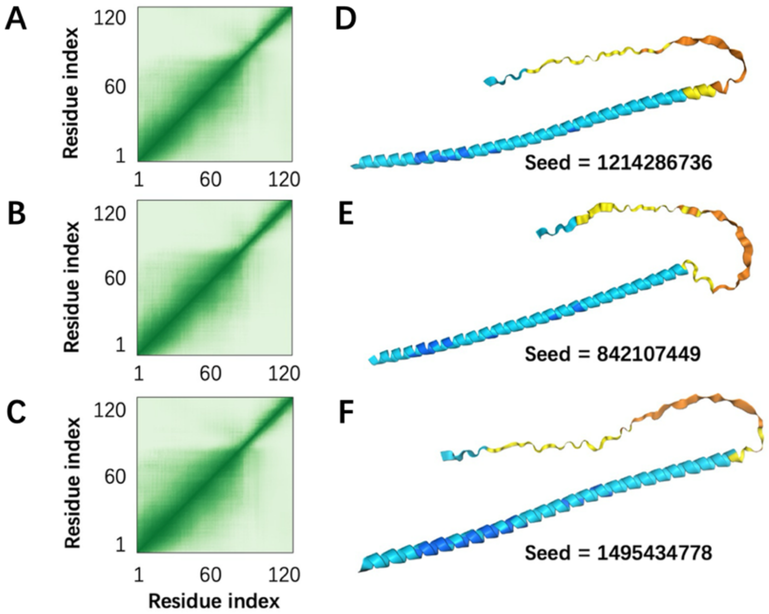

Fig. S32.

Prediction of αSyn monomer structure by AlphaFold 2. (A-C) Confidence scores of residue-residue interaction predictions in three individual prediction runs. **(D-F)** αSyn monomer structures predicted by three individual prediction runs. Different color represents different degree of local confidence (plDDT): dark blue, sky blue, yellow, and orange color respectively corresponds to plDDT within [90,100], [70,90), [50,70), and [0,50).

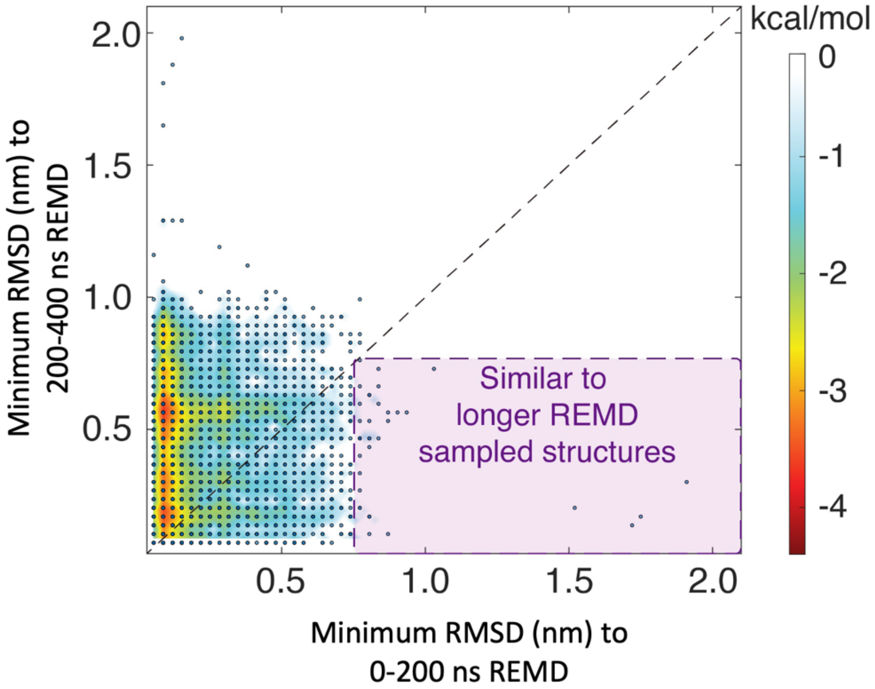

Fig. S33.

RMSD of each VAE-generated αSyn structure. The x and y axes respectively correspond to the RMSD of generated structure with respect to its most similar structure in the first and second half of our REMD simulation.

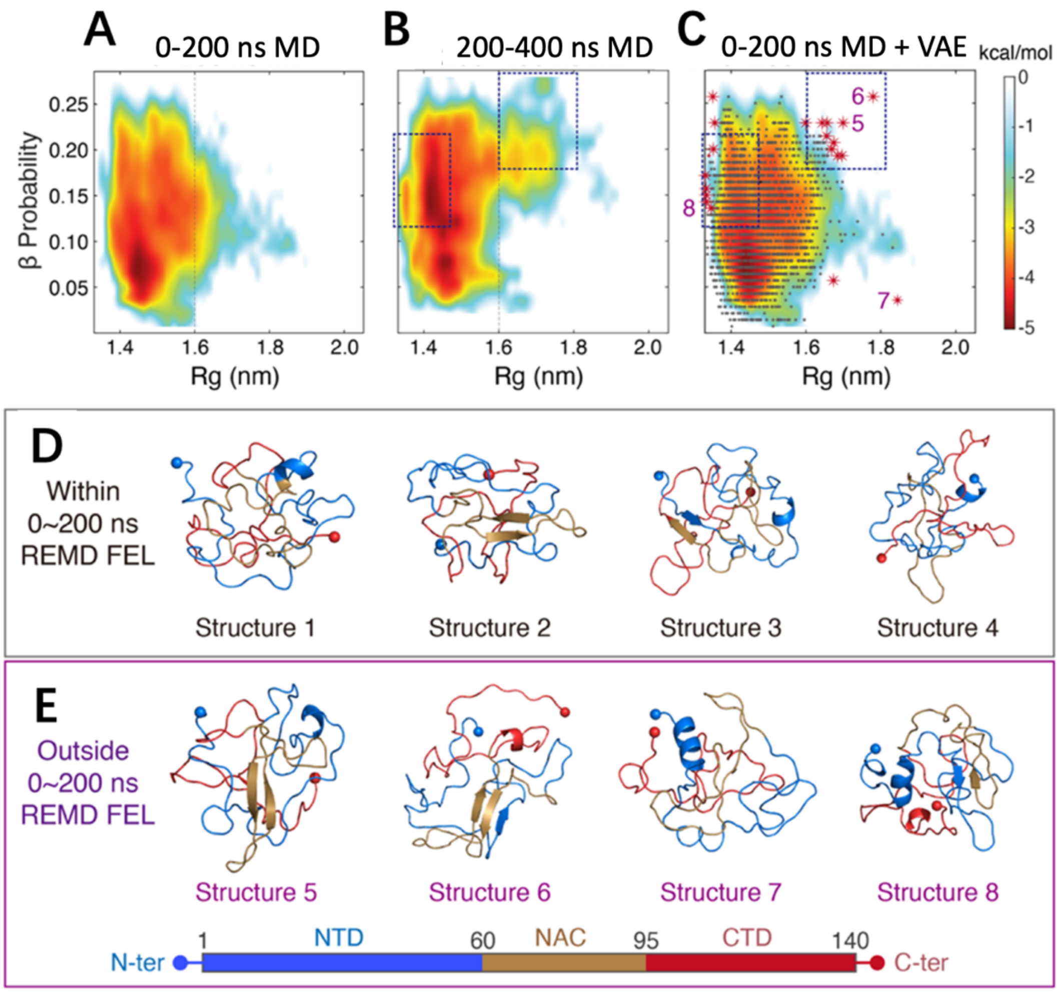

Fig. S34.

Characterization to the VAE-generated structures. (A-B) The free energy landscape (FEL) of αSyn conformations sampled by (A) the first half and (B) the second half of our REMD simulation. **(C)** Projection of VAE-generated structures on the FEL the first half of REMD simulation. **(D-E)** Snapshots of representative structures generated by VAE which locate (D) within and (E) outside the FES of the first half of REMD simulation.

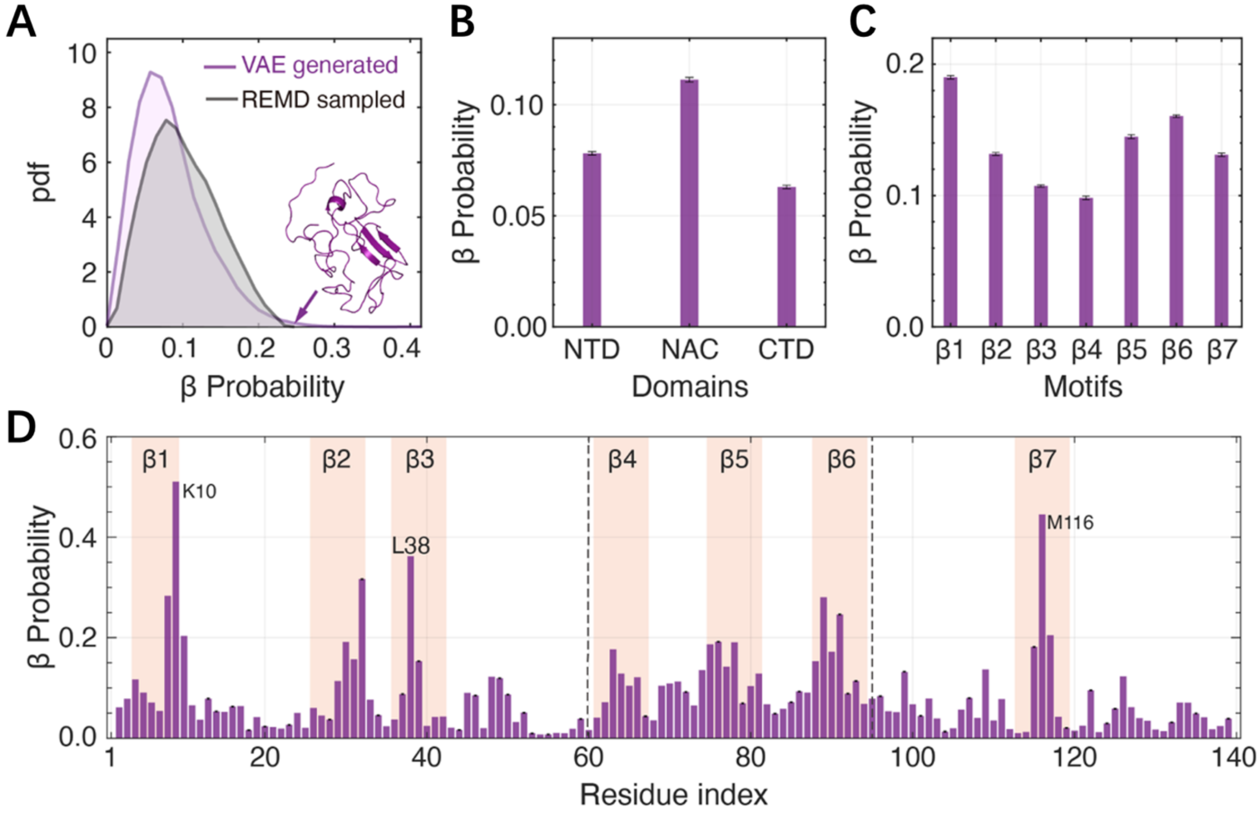

Fig. S35.

β structure analysis of the VAE generated αSyn structures. (A) PDF for β probabilities of αSyn structures sampled by REMD and those generated by VAE. **(B-C)** β probability of (B) NTD, NAC, CTD domains and (C) seven high-β propensity motifs. **(D)** β probability of each residue in αSyn structures generated by VAE.

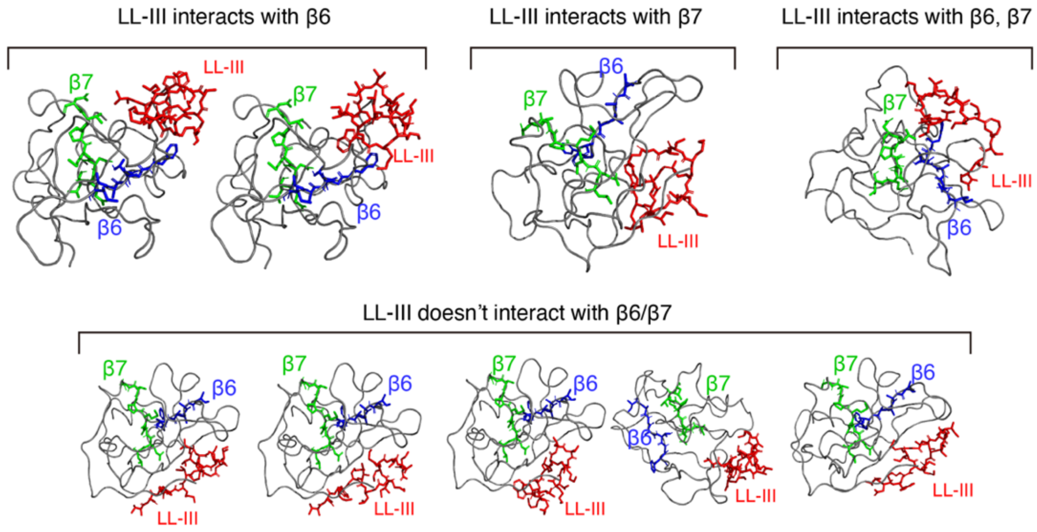

Fig. S36.
Snapshots to the complex of αSyn monomer and LL-III predicted by nine independent docking runs.

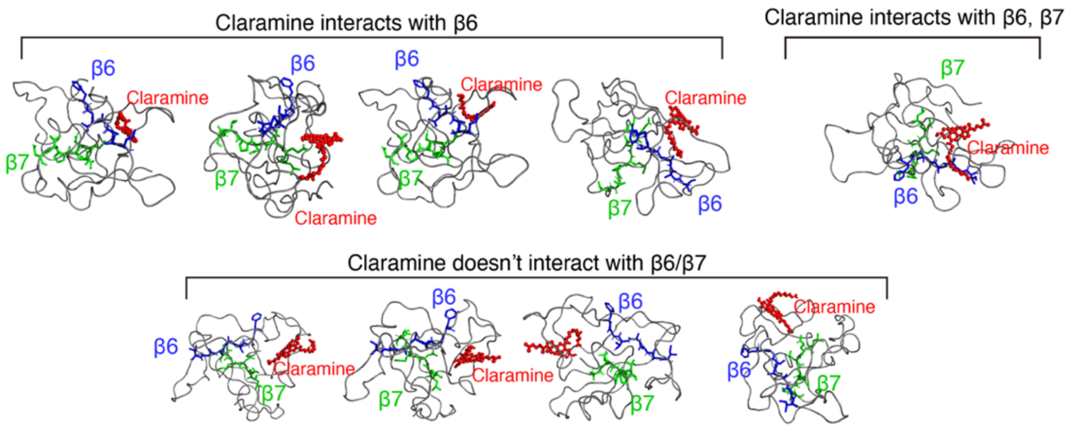

Fig. S37.
Snapshots to the complex of αSyn monomer and claramine predicted by nine independent docking runs.

Table S1.

Details of the all-atom and CG simulations.

Protein	αSyn (WT)	αSyn (WT)	αSyn (WT)	αSyn (Δβ6)	αSyn (Δβ7)	αSyn (WT)
System	Monomer	Condensate	Phase coexistence			
Simulation method	REMD	MD	MD			
Protein model	All-atom	All-atom	Coarse-grained			
Force field	Amber 99SB-ILDN		HPS			HPS-T
Initial structure	*Proc Natl Acad Sci* **2018**, *115* (21), E4758. [6]	From monomer REMD and VAE	From condensation MD			
Chain number	1	60	216	216	216	216
Number of atoms/beads	37667	1,024,660	30,240	30,240	30,240	30,240
Simulation time	400 ns	3 μs	4 μs	4 μs	4 μs	4 μs
Number of replicas	60	/	/	/	/	/
Number of simulations	1	1	9	9	9	26
Temperature	308-410 K	310 K	300-380 K	300-380 K	300-380 K	280-380 K

Table S2.

Temperature list of the 60 replicas in the REMD simulation.

Replica index	Temperature (K)	Replica index	Temperature (K)	Replica index	Temperature (K)
1	308.0	21	339.9	41	374.3
2	309.5	22	341.6	42	376.1
3	311.1	23	343.2	43	377.9
4	312.6	24	344.9	44	379.8
5	314.2	25	346.6	45	381.6
6	315.8	26	348.3	46	383.4
7	317.3	27	350.0	47	385.2
8	318.9	28	351.7	48	387.1
9	320.5	29	353.4	49	388.9
10	322.0	30	355.1	50	390.8
11	323.6	31	356.8	51	392.6
12	325.2	32	358.6	52	394.5
13	326.8	33	360.3	53	396.4
14	328.5	34	362.0	54	398.3
15	330.1	35	363.7	55	400.2
16	331.7	36	365.4	56	402.1
17	333.3	37	367.2	57	404.0
18	335.0	38	369.0	58	405.9
19	336.6	39	370.7	59	407.8
20	338.3	40	372.5	60	409.8

Table S3. Reported radius of gyration (Rg) values for monomeric αSyn from previous experimental studies. Note that values derived from NMR studies correspond to hydrodynamic radii, which are generally larger than the radius of gyration. We thus add a "<" symbol before these values.

Rg (nm)	Method	Remark	Reference
1.8-6.0	SAXS	NaH_2PO_4/Na_2HPO_4 buffer	*Structure* **2014**, 22 (2), 238−249. [19a]
< 2.2	NMR	1M glucose	*Protein Science* **2008**, 10 (11), 2195−2199. [19b]
< 2.6	NMR	Without glucose	
2.72	SAXS	Ammonium acetate buffer	*Scientific Reports* **2016**, 6 (1), 30473. [19c]
3.59	SEC-SAXS	Tris buffer	
4.27	SAXS	Tris buffer	

Table S4.

Helix and β-sheet contents of full-length αSyn and three αSyn domains. Displayed are experimentally derived values and results from our REMD simulation.

	**Full-length αSyn**			**NTD**	**NAC**	**CTD**
**Methods**	CD [20b]	FTIR [20a]	REMD	REMD	REMD	REMD
**β-sheet**	11%	15.6%	12.9%	12.4%	18.7%	9.2%
**Helix**	2%	/	9.0%	11.9%	8.2%	6.6%

Table S5.

The four helix-rich and seven β-sheet-rich motifs identified through all-atom REMD simulation on αSyn monomer.

Type	Name	Sequence	Location
**Helix-rich motifs**	H1	$_{18}$AAEKTKQ$_{24}$	NTD
	H2	$_{41}$GSKTKEG$_{47}$	NTD
	H3	$_{55}$VAEKTKE$_{61}$	NTD, NAC
	H4	$_{128}$PSEEGYQ$_{134}$	CTD
**β-sheet-rich motifs**	β1	$_{4}$FMKGLSK$_{10}$	NTD
	β2	$_{26}$VAEAAGK$_{32}$	NTD
	β3	$_{36}$GVLYVGS$_{42}$	NTD
	β4	$_{61}$EQVTNVG$_{67}$	NAC
	β5	$_{75}$TAVAQKT$_{81}$	NAC
	β6	$_{88}$IAAATGF$_{94}$	NAC
	β7	$_{113}$LEDMPVD$_{119}$	CTD

Table S6.

The diffusion constant of the αSyn condensate within four time windows in the million-atom MD simulation.

**Time window (μs)**	1.0-1.5	1.5-2.0	2.0-2.5	2.5-3.0
**Diffusion constant (10^{-12} m^2/s)**	2.01 ± 0.12	1.39 ± 0.08	0.89 ± 0.10	0.76 ± 0.11

Movie captions

Movie S1.

A local scene of the αSyn condensate comprising cross-linked fibrous chains interspersed with substantial quantities of water molecules.

Movie S2.

Time evolution of the conformation of a representative αSyn chain during the condensation and aging processes.

Movie S3.

The phase separation simulation of full-length wild type αSyn at eight temperature points.

Movie S4.

The phase separation simulation of full-length wild type αSyn and its motif-deleted variants $\alpha Syn_{\Delta\beta6}$ and $\alpha Syn_{\Delta\beta7}$ at physiological temperature.

References.

[1] D. Van Der Spoel, E. Lindahl, B. Hess, G. Groenhof, A. E. Mark, H. J. Berendsen, *J Comput Chem* **2005**, *26* (16), 1701.

[2] K. Lindorff-Larsen, S. Piana, K. Palmo, P. Maragakis, J. L. Klepeis, R. O. Dror, D. E. Shaw, *Proteins* **2010**, *78* (8), 1950.

[3] D. van der Spoel, M. M. Seibert, *Phys Rev Lett* **2006**, *96* (23), 238102.

[4] A. Patriksson, D. van der Spoel, *Phys Chem Chem Phys* **2008**, *10* (15), 2073.

[5] a) A. P. Longhini, A. DuBose, S. Lobo, V. Vijayan, Y. Bai, E. K. Rivera, J. Sala-Jarque, A. Nikitina, D. C. Carrettiero, M. T. Unger, O. R. Sclafani, V. Fu, E. R. Beckett, M. Vigers, L. Buee, I. Landrieu, S. Shell, J. E. Shea, S. Han, K. S. Kosik, *Proc Natl Acad Sci U S A* **2024**, *121* (15), e2320456121; b) X. Dong, S. Bera, Q. Qiao, Y. Tang, Z. Lao, Y. Luo, E. Gazit, G. Wei, *J Phys Chem Lett* **2021**, *12* (10), 2576; c) F. Jiang, Y. D. Wu, *J Am Chem Soc* **2014**, *136* (27), 9536; d) Y. Chen, X. Sun, Y. Tang, Y. Tan, C. Guo, T. Pan, X. Zhang, J. Luo, G. Wei, *Small* **2024**, 2406429; e) M. P. Vigers, S. Lobo, S. Najafi, A. Dubose, K. Tsay, P. Ganguly, A. P. Longhini, Y. Jin, S. K. Buratto, K. S. Kosik, M. S. Shell, J. E. Shea, S. Han, *Proc Natl Acad Sci U S A* **2025**, *122* (18), e2421391122.

[6] P. Robustelli, S. Piana, D. E. Shaw, *Proc Natl Acad Sci U S A* **2018**, *115* (21), E4758.

[7] T. Bartels, J. G. Choi, D. J. Selkoe, *Nature* **2011**, *477* (7362), 107.

[8] T. Darden, D. York, L. Pedersen, *J Chem Phys* **1993**, *98* (12), 10089.
[9] M. Parrinello, A. Rahman, *J Appl Phys* **1981**, *52* (12), 7182.
[10] R. M. Regy, G. L. Dignon, W. Zheng, Y. C. Kim, J. Mittal, *Nucleic Acids Res* **2020**, *48* (22), 12593.
[11] U. Kapoor, Y. C. Kim, J. Mittal, *J Chem Theory Comput* **2024**, *20* (4), 1717.
[12] H. I. Ingolfsson, A. Rizuan, X. Liu, P. Mohanty, P. C. T. Souza, S. J. Marrink, M. T. Bowers, J. Mittal, J. Berry, *Biophys J* **2023**, *122* (22), 4370.
[13] N. Galvanetto, M. T. Ivanovic, A. Chowdhury, A. Sottini, M. F. Nuesch, D. Nettels, R. B. Best, B. Schuler, *Nature* **2023**, *619* (7971), 876.
[14] S. Mukherjee, L. V. Schafer, *Nat Commun* **2023**, *14* (1), 5892.
[15] G. Krainer, T. J. Welsh, J. A. Joseph, J. R. Espinosa, S. Wittmann, E. de Csillery, A. Sridhar, Z. Toprakcioglu, G. Gudiskyte, M. A. Czekalska, W. E. Arter, J. Guillen-Boixet, T. M. Franzmann, S. Qamar, P. S. George-Hyslop, A. A. Hyman, R. Collepardo-Guevara, S. Alberti, T. P. J. Knowles, *Nat Commun* **2021**, *12* (1), 1085.
[16] J. Wen, L. Hong, G. Krainer, Q. Q. Yao, T. P. J. Knowles, S. Wu, S. Perrett, *J Am Chem Soc* **2021**, *143* (33), 13056.
[17] S. Y. Mandaci, M. Caliskan, M. F. Sariaslan, V. N. Uversky, O. Coskuner-Weber, *Chem Biol Drug Des* **2020**, *96* (1), 659.
[18] G. H. Zerze, W. Zheng, R. B. Best, J. Mittal, *J Phys Chem Lett* **2019**, *10* (9), 2227.
[19] a) M. Schwalbe, V. Ozenne, S. Bibow, M. Jaremko, L. Jaremko, M. Gajda, Malene R. Jensen, J. Biernat, S. Becker, E. Mandelkow, M. Zweckstetter, M. Blackledge, *Structure* **2014**, *22* (2), 238; b) A. S. Morar, A. Olteanu, G. B. Young, G. J. Pielak, *Protein Science* **2008**, *10* (11), 2195; c) K. Araki, N. Yagi, R. Nakatani, H. Sekiguchi, M. So, H. Yagi, N. Ohta, Y. Nagai, Y. Goto, H. Mochizuki, *Sci Rep* **2016**, *6*, 30473.
[20] a) V. N. Uversky, J. Li, A. L. Fink, *J Biol Chem* **2001**, *276* (14), 10737; b) A. Rekas, R. B. Knott, A. Sokolova, K. J. Barnham, K. A. Perez, C. L. Masters, S. C. Drew, R. Cappai, C. C. Curtain, C. L. Pham, *Eur Biophys J* **2010**, *39* (10), 1407.
[21] a) L. Gadhe, A. Sakunthala, S. Mukherjee, N. Gahlot, R. Bera, A. S. Sawner, P. Kadu, S. K. Maji, *Biophys Chem* **2022**, *281*, 106736; b) M. C. Hardenberg, T. Sinnige, S. Casford, S. T. Dada, C. Poudel, E. A. Robinson, M. Fuxreiter, C. F. Kaminksi, G. S. Kaminski Schierle, E. A. A. Nollen, C. M. Dobson, M. Vendruscolo, *J Mol Cell Biol* **2021**, *13* (4), 282; c) M. Takamuku, T. Sugishita, H. Tamaki, L. Dong, M. So, T. Fujiwara, Y. Matsuki, *Neurochem Int* **2022**, *157*, 105345; d) S. Ray, N. Singh, R. Kumar, K. Patel, S. Pandey, D. Datta, J. Mahato, R. Panigrahi, A. Navalkar, S. Mehra, L. Gadhe, D. Chatterjee, A. S. Sawner, S. Maiti, S. Bhatia, J. A. Gerez, A. Chowdhury, A. Kumar, R. Padinhateeri, R. Riek, G. Krishnamoorthy, S. K. Maji, *Nat Chem* **2020**, *12* (8), 705.
[22] a) D. Dibenedetto, G. Rossetti, R. Caliandro, P. Carloni, *Biochemistry* **2013**, *52* (38), 6672; b) A. Sanjeev, V. S. K. Mattaparthi, *J Biomol Struct Dyn* **2018**, *36* (9), 2224; c) D. Huang, C. Guo, *J Chem Inf Model* **2023**, *63* (15), 4803; d) Z. Yang, Y. Yao, Y. Zhou, X. Li, Y. Tang, G. Wei, *Int J Biol Macromol* **2023**, *230*, 123194.
[23] a) J. N. Rao, Y. E. Kim, L. S. Park, T. S. Ulmer, *J Mol Biol* **2009**, *390* (3), 516; b) C. A. Waudby, C. Camilloni, A. W. Fitzpatrick, L. D. Cabrita, C. M. Dobson, M. Vendruscolo, J. Christodoulou, *PLoS One* **2013**, *8* (8), e72286; c) Y. Pustovalova, M. Mayzel, V. Y. Orekhov, *Angew Chem Int Ed Engl* **2018**, *57* (43), 14043.
[24] C. Camilloni, A. De Simone, W. F. Vranken, M. Vendruscolo, *Biochemistry* **2012**, *51* (11), 2224.
[25] T. Graen, R. Klement, A. Grupi, E. Haas, H. Grubmüller, *ChemPhysChem* **2018**, *19* (19), 2507.
